# Green-synthesized zinc and iron nanoparticles enhance basil drought tolerance via antioxidant regulation

**DOI:** 10.1186/s12870-026-09935-3

**Published:** 2026-09-12

**Authors:** Ibrahim Selvikaya, Rufayi Karataş, Murat Karakuş, Hilal Yilmaz, Fatih Demirel, Emrah Güler, Yusuf Tutar, Abdurrahim Yilmaz

**Affiliations:** 1 Department of Field Crops, Faculty of Agriculture, Bolu Abant Izzet Baysal University, Bolu, 14030 Türkiye; 2 Department of Field Crops, Faculty of Agriculture, Atatürk University, Erzurum, 25240 Türkiye; 3 Department of Plant and Animal Production, Seben Izzet Baysal Vocational School, Bolu Abant Izzet Baysal University, Bolu, 14750 Türkiye; 4 Plant and Animal Production Program, Izmit Vocational School, Kocaeli University, Kocaeli, 41285 Türkiye; 5 Department of Agricultural Biotechnology, Faculty of Agriculture, Igdir University, Igdir, 76000 Türkiye; 6 Department of Horticulture, Faculty of Agriculture, Bolu Abant Izzet Baysal University, Bolu, 14030 Türkiye; 7 Division of Medicinal Biochemistry, Department of Basic Medical Sciences, Faculty of Medicine, Recep Tayyip Erdogan University, Rize, 53020 Türkiye

**Keywords:** *Ocimum basilicum*, Drought stress, Green-synthesized nanoparticles, Antioxidant defense, Redox regulation, Stress physiology

## Abstract

**Background:**

Drought is a major abiotic constraint that disrupts growth, physiology, and metabolic balance in medicinal and aromatic plants. This study examined how green-synthesized zinc (Zn) oxide and iron (Fe) oxide nanoparticles (NPs) influence drought tolerance in basil (*Ocimum basilicum* L.). A factorial greenhouse experiment evaluated two irrigation regimes (100% and 50% field capacity, FC) combined with four foliar treatments: control, Zn (100 mg L⁻^1^), Fe (100 mg L⁻^1^), and Zn + Fe (50 + 50 mg L⁻^1^), which represent agronomically realistic and field-applicable concentrations. Nanoparticles biosynthesized using sage (*Salvia officinalis*) extract were characterized by scanning electron microscopy/energy dispersive X-ray spectroscopy (SEM/EDS).

**Results:**

Under drought conditions, NP supplementation mitigated growth reductions by increasing plant height by up to 26.7%, leaf number by 30.6%, and biomass by 22.6% compared to untreated drought controls. The combined Zn + Fe treatment elicited trait-specific responses, particularly increasing phenolic and flavonoid contents by 53% and 48%, respectively, and notably enhancing catalase (CAT) and ascorbate peroxidase (APX) activities by 204% and 86% relative to drought controls. Consequently, oxidative stress markers (malondialdehyde [MDA], hydrogen peroxide [H₂O₂]) were reduced by nearly 50%. Zn primarily enhanced non-enzymatic antioxidant capacity by increasing cupric reducing antioxidant capacity [CUPRAC], ferric reducing antioxidant power [FRAP], and 2,2-diphenyl-1-picrylhydrazyl [DPPH] by 135%, 48%, and 17%, respectively, whereas Fe supported enzymatic detoxification through a 27% increase in superoxide dismutase (SOD) activation compared to drought controls. Correlation analysis showed strong associations among antioxidant capacity, pigment levels, and growth traits, and principal component analysis (PCA) distinguished Zn + Fe-treated plants within antioxidant-rich and high-biomass clusters, indicating coordinated redox regulation.

**Conclusions:**

Foliar nano-micronutrition with green-synthesized Zn NPs and Fe NPs strengthened antioxidant defenses, stabilized pigments, and improved growth resilience under drought, providing physiological and biochemical evidence of nanoparticle-mediated modulation of stress responses.

**Supplementary Information:**

The online version contains supplementary material available at https://doi.org/10.1186/s12870-026-09935-3.

## Introduction

Global climate change has become one of the most pressing environmental constraints on agriculture, with irregular precipitation, rising temperatures, and reduced water availability intensifying abiotic stresses and threatening food security [1–4]. Among these stresses, drought is particularly detrimental, impairing water uptake, photosynthesis, respiration, hormonal balance, and nutrient transport, ultimately leading to growth retardation, leaf senescence, and quality losses [5–8]. Beyond visible symptoms, prolonged drought disrupts redox homeostasis and suppresses secondary metabolite synthesis—both essential for plant resilience [9, 10].

Water deficit is closely linked to oxidative stress, as impaired stomatal conductance and reduced photosynthetic activity promote excessive accumulation of reactive oxygen species (ROS) in chloroplasts, mitochondria, and peroxisomes [11, 12]. These ROS interact with lipids, proteins, and nucleic acids, causing oxidative damage typically monitored by markers such as H₂O₂ and MDA. To counter such damage, plants activate enzymatic (SOD, CAT, APX) and non-enzymatic (phenolics, flavonoids, carotenoids) antioxidant systems [13–15]. The efficiency of these defenses depends on coordinated redox signaling integrating enzymes, secondary metabolites, and photosynthetic pigments into a unified antioxidant network [16–18].

Nanotechnology has recently emerged as a promising strategy to enhance plant tolerance to abiotic stresses [19, 20]. Owing to their nanoscale size and high surface reactivity, nanoparticles (NPs) exhibit distinct physicochemical and biological interactions that can modulate physiological and metabolic processes [21–23]. Nano-fertilizers offer controlled nutrient delivery and improved uptake efficiency, reducing reliance on conventional fertilizers while supporting sustainable production [24–27]. Among micronutrient-based NPs, zinc (Zn) and iron (Fe)—two elements whose widespread deficiencies critically threaten global nutritional security [28]— stand out and, functionally, play vital roles in enzyme activation, chlorophyll biosynthesis, respiration, and redox regulation [29–32]. Emerging evidence suggests that nano-Zn and nano-Fe oxide may act as redox modulators, enhancing ROS-scavenging capacity and stabilizing photosynthetic processes through synergistic effects across antioxidant pathways [33–35].

Medicinal and aromatic plants are increasingly studied for their stress-responsive bioactive profiles [36–38]. The growing reliance on natural resources within the healthcare industry has significantly enhanced the value and importance of medicinal and aromatic plant species [39–42]. Basil (*Ocimum basilicum* L.), valued for its essential oils, phenolics, and flavonoids with diverse pharmacological properties [43–46], is particularly sensitive to drought, which compromises both yield and phytochemical quality [47–50]. Developing sustainable approaches to improve basil’s drought resilience is therefore of growing importance.

Within this context, we hypothesized that the foliar application of green-synthesized Zn NPs and Fe NPs—particularly in combination (Zn + Fe NPs)—would synergistically mitigate drought-induced oxidative damage in basil by upregulating coordinated antioxidant defenses and maintaining pigment stability. To test this hypothesis, the present study examines how green-synthesized Zn NPs and Fe NPs influence redox regulation, antioxidant coordination, and pigment stability in drought-stressed basil. By integrating morphological, physiological, and biochemical assessments with correlation and PCA analyses, the study aims to elucidate systemic mechanisms underlying nanoparticle-induced drought tolerance. This integrative perspective aligns with current priorities in plant systems biology and contributes to sustainable production strategies for medicinal and aromatic plants.

## Materials and methods

### Experimental area, plant material, and soil properties

The pot experiment was conducted between June and August 2024 in a semi-controlled greenhouse at the Department of Horticulture, Faculty of Agriculture, Bolu Abant İzzet Baysal University, Türkiye. A broad-leaved basil cultivar, “Dino,” obtained from AG Tohum Company, was used as plant material. The growth medium consisted of soil collected from the faculty’s experimental fields. The soil was air-dried, passed through a 5 mm sieve, and mixed with Kekkilä DSM Series peat at a 1:1 (v/v) ratio to obtain a 50:50 substrate. The mixture was placed into 1.5-L pots in equal proportions. The physical and chemical characteristics of the soil are provided in Table 1.

**Table 1:** Physical and chemical properties of the soil used in the experiment

Parameter	Value	Classification	Analytical method
Saturation (%)	68.31	Clay-Loam	TS 8333 (Air Dry, 10%)
pH	7.25	Neutral	Yurdakul (2018)
Total salt (%)	0.09	Non-saline	TS 8334 (in Mud)
Lime (%)	1.44	Calcareous	TS EN ISO 10693
Organic matter (%)	7.39	High	TS 8336
Available phosphorus (kg da^−1^)	48.57	Moderate to Very High	Bray and Kurtz (1945)
Available potassium (kg da^−1^)	294.51	High	Olsen (1954)—Absorption

### Green synthesis of iron nanoparticles (Fe NPs)

Iron oxide nanoparticles were synthesized from *Salvia officinalis* leaf extract following a modified method of Wang et al. [51]. Fresh sage leaves (100 g), collected from Bolu-Çampınar, were ground and heated in 500 mL Milli-Q water at 80 °C for 1 h. After cooling for 1 h, the extract was filtered through Whatman No.1 paper. Separately, 16.23 g FeCl₃ (Sigma-Aldrich) was dissolved in 1.0 L Milli-Q water to prepare a 0.1 M solution. The leaf extract and FeCl₃ solution were combined at a 1:2 (v/v) ratio, and the pH was slowly adjusted to 11 using 0.1 M NaOH while the mixture was stirred at 80 °C for an additional 10 min.

Synthesis was visually confirmed by the solution turning dark brown. The mixture was cooled to room temperature, and the resulting nanoparticle precipitate was collected by centrifugation at 4000 rpm. To remove unreacted precursors and impurities, the collected precipitate was washed three times by sequentially resuspending it in distilled water and re-centrifuging. The collected Fe NPs were dried at 70 °C for 24 h, ground into fine powder using a porcelain mortar, and stored in amber glass vials at + 4 °C until further use.

### Green synthesis of zinc nanoparticles (Zn NPs)

Zinc oxide NPs were biologically synthesized from *Salvia officinalis* leaves following Abomuti et al. [52] with minor modifications. For the preparation of the aqueous leaf extract, 5 g of finely ground dried sage leaves were mixed with 200 mL distilled water in a 250 mL beaker, heated on a magnetic stirrer at 60 °C for ~ 2 h, and stored overnight in an airtight glass bottle. The mixture was then filtered through Whatman No.1 paper, and the filtrate was refrigerated until use.

For Zn NP synthesis, 100 mL of 0.2 M Zn(NO₃)₂ solution was placed in a 500 mL beaker and stirred at 50 °C for 10 min. The sage extract (100 mL), preheated to the same temperature, was added to the zinc nitrate solution. The reaction was maintained at 50 °C for 2 h to promote electrostatic interactions between Zn^2^⁺ ions and phytochemicals. A light-yellow color change indicated the onset of Zn particle nucleation and putative phytochemical-mediated capping. As nucleation progressed, stabilization occurred at a naturally low pH of ~ 2.0.

The pH gradually increased to 12 by dropwise addition of freshly prepared 2.0 M NaOH under continuous stirring. Formation of a light-yellow precipitate during heating at 50 °C indicated the successful synthesis of Zn NPs. The precipitate was collected by centrifugation at 10,000 rpm for 20 min, washed multiple times with ethanol and distilled water, and dried at 80 °C for 24 h. The dried material was finely ground in a porcelain mortar to obtain uniform Zn nanopowder for subsequent analyses and characterization.

### Characterization of nanoparticles

**Fig. 1:**
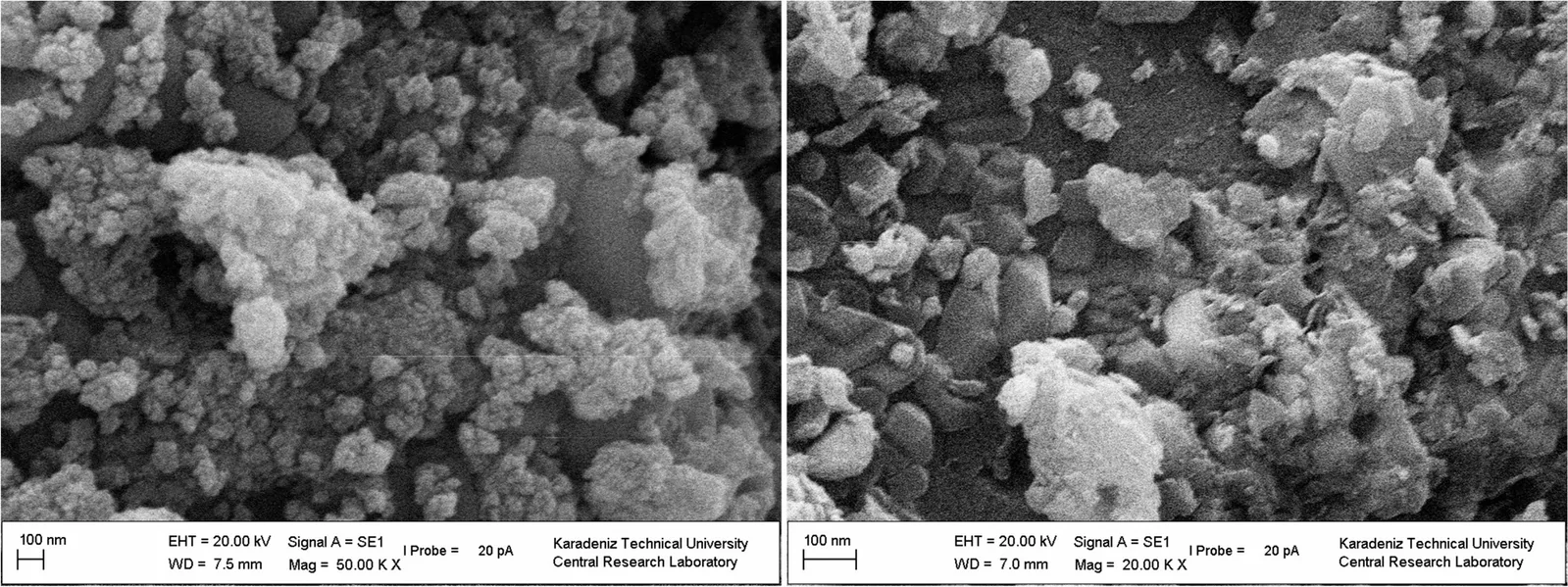
SEM images illustrating the surface morphology of biosynthesized zinc oxide (Zn; left) and iron oxide (Fe; right) nanoparticles obtained from *Salvia officinalis* extract

The morphology of the synthesized zinc and iron nanoparticles was examined using scanning electron microscopy (SEM; ZEISS EVO LS 10) at the Central Laboratory of Karadeniz Technical University (Fig. 1). SEM images revealed predominantly spherical particles with slight agglomeration. Based on the 100 nm scale bar, particle diameters ranged from ~ 50 to 250 nm, confirming that the green-synthesized materials exhibited submicron to nanoscale dimensions relevant to biological activity. While particles > 100 nm partially exceed strict conventional nanoscale definitions, broad size distributions and agglomeration are highly characteristic of plant-mediated green synthesis. In the context of bulk agricultural foliar applications, plants are typically exposed to these complex, agglomerated submicron suspensions rather than mono-dispersed nanoparticles.

**Fig. 2:**
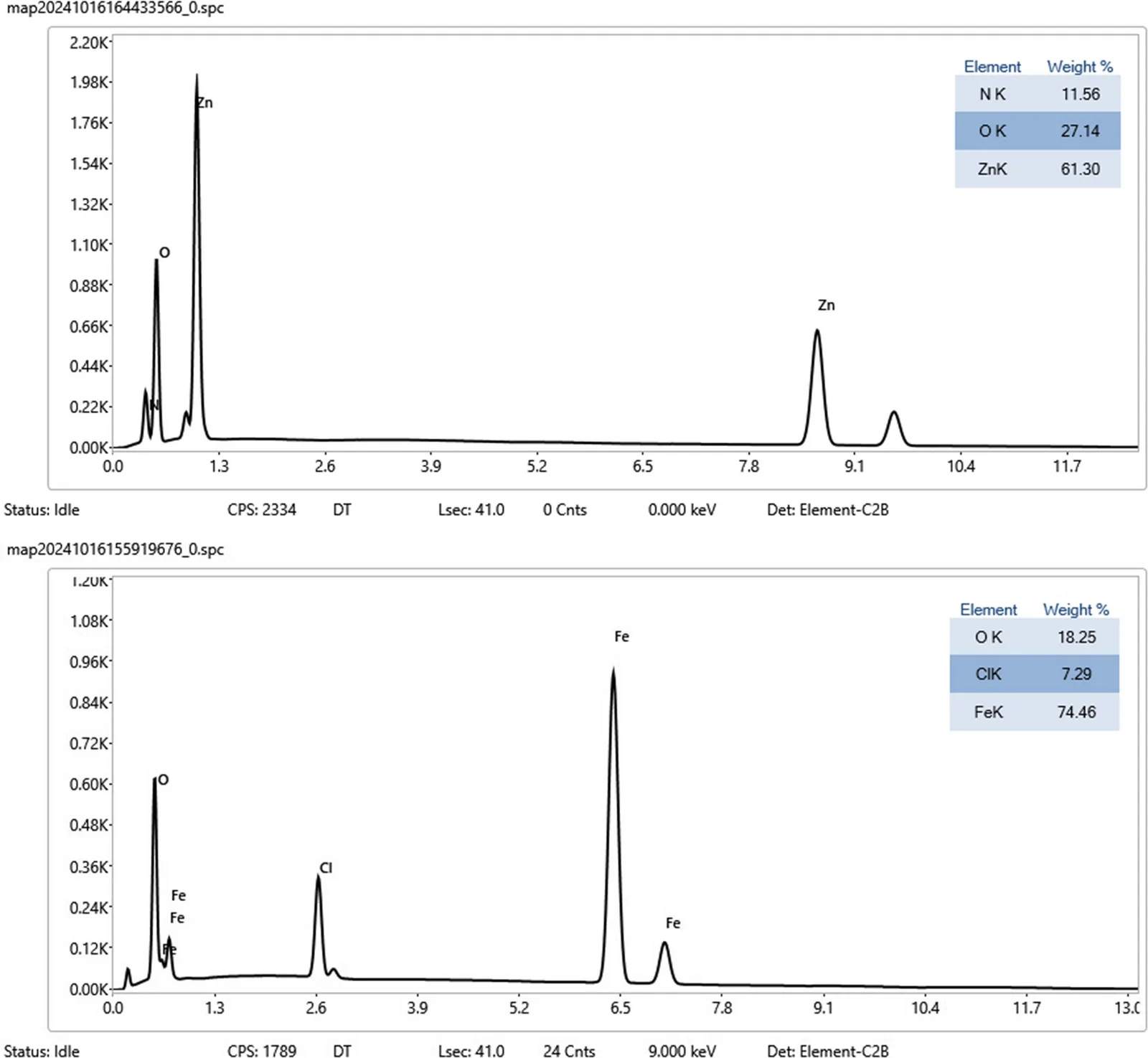
Elemental composition analysis of biosynthesized Zn (top) and Fe (bottom) NPs using EDS

Elemental composition was determined using Energy Dispersive X-ray Spectroscopy (EDS) (Fig. 2). For Zn NPs, the spectra indicated the presence of zinc (61.30%), oxygen (27.14%), and nitrogen (11.56%). The combined Zn and O contents corresponded to approximately 87.86%. For Fe NPs, EDS revealed iron (74.46%), oxygen (18.25%), and chlorine (7.29%), corresponding to ~ 92.71% Fe and O.

While multiple sequential washing steps (centrifugation with distilled water and ethanol) were performed to remove the bulk of unreacted precursors, the residual nitrogen and chlorine detected via EDS likely represent precursor ions (NO₃⁻ and Cl⁻) physically trapped within the bulky phytochemical capping matrix of the sage extract. Because plant extracts contain unquantified, complex mixtures of reducing agents, exact stoichiometric conversion rates and absolute yields cannot be definitively calculated as in purely chemical synthesis. In the context of green agricultural nanotechnology, producing highly purified, analytical-grade nanomaterials is neither cost-effective nor practically necessary. Instead, these 'crude' biogenic nanopowders—which retain trace precursor elements tightly bound within the bioactive phytochemical corona—are typically utilized directly for foliar application, faithfully representing scalable and realistic agricultural biostimulant formulations.

### Experimental design, irrigation and foliar application management

Basil seedlings were transplanted into pots in June, with one plant per pot, and grown under semi-controlled greenhouse conditions. During the experimental period, the plants were exposed to natural sunlight (approximate 14/10 h light/dark cycle). Greenhouse temperature and relative humidity were regulated using an air conditioning system, maintaining day/night temperatures at approximately 28–36 °C/18–22 °C and a relative humidity of 55–65%. The experiment followed a factorial design consisting of four NP treatments—Control (no application), Zn NPs (100 mg L⁻^1^), Fe NPs (100 mg L⁻^1^), and Zn + Fe NPs (50 mg L⁻^1^ + 50 mg L⁻^1^)—and two irrigation regimes (100% and 50% FC), yielding 8 treatment combinations. Each treatment group was established with three biological replicates, where each replicate consisted of a pooled subset of three individual potted plants. Therefore, while a total of 72 plants were cultivated across the study (9 plants contributing material per treatment), the statistical unit of replication entered into the analyses was *n* = 3 pooled samples per treatment. The singular application doses for Zn NPs and Fe NPs followed Khan et al. [53] and Kilic et al. [54], respectively. Crucially, the combined treatment was intentionally formulated as a 50:50 ratio (50 mg L⁻^1^ Zn NPs + 50 mg L⁻^1^ Fe NPs) to strictly maintain the total applied nano-metal concentration at 100 mg L⁻^1^. This specific design ensured that any superior physiological responses observed under the combined treatment could be definitively attributed to the synergistic interaction of the two distinct metals, rather than simply being an artifact of doubling the total metal load (which would also risk phytotoxicity).

Pots were irrigated with distilled water to maintain predetermined FC levels. Field capacity was determined by saturating the soil and allowing free drainage for 24 h following Özel et al. [55]. Every 2–3 days, pots were weighed to assess water loss from evapotranspiration, and distilled water was added to restore the target moisture.

The 50% FC treatment was initiated gradually after a three-week post-transplant adaptation period (when the plants were approximately 51 days old). Four days after establishing the water deficit, foliar applications of Nano-Zn, Nano-Fe, and Nano Fe + Zn were performed using a hand-held sprayer. A total of four foliar sprays were applied at approximately 4-day intervals over the 15-day deficit period. Pots were covered during spraying to prevent cross-contamination into the soil, and applications of Zn NPs, Fe NPs, and Zn + Fe NPs were conducted in a wind-free environment. Following the completion of the 15-day nanoparticle application period, the plants were maintained under their respective irrigation regimes (100% and 50% FC) for an additional ~ 15 days to allow for full metabolic integration and maturation. Finally, after a total 60-day post-transplant growth period, all basil plants were harvested simultaneously in August.

### Physical and morphological analyses

Before harvest, plant height (cm) and leaf number were recorded. Plants were then removed from pots, and roots were separated using scissors. Fresh weights of whole plants and leaves (g plant⁻^1^) were measured using an analytical balance (± 0.0001 g). Plant height, leaf number, plant weight, and leaf weight were used as the main growth parameters.

### Tissue preparation and biochemical normalization

To prevent the degradation of oxidation-sensitive secondary metabolites and to ensure the in vivo accuracy of transient enzymatic activities, all biochemical assays were conducted using fresh leaf tissues that were processed immediately upon harvest. Consequently, all subsequent spectrophotometric quantifications for pigments, non-enzymatic antioxidants, and stress enzymes were normalized to a fresh weight basis.

### Total phenolic and flavonoid contents

Total phenolic content (TPC) was determined following Waterhouse [56] with minor modifications. For the extraction of phenolic compounds, 1000 mg of leaf tissue was homogenized in 10 mL of 80% methanol. The homogenate was centrifuged at 13.000 rpm for 5 min at 4 °C, and the resulting supernatant was collected as the methanolic extract. A reaction mixture containing 1.6 mL distilled water, 50 µL methanolic extract, and 50 µL Folin–Ciocalteu reagent was prepared. Subsequently, 300 µL of 7% calcium carbonate solution was added, vortexed, and incubated in the dark at room temperature for 2 h. Absorbance was measured at 760 nm using a UV–Vis spectrophotometer (SP-UV1100, DLAB, Beijing, China). Standard gallic acid solutions (0.5–6 mM; R^2^ = 0.99) were used for calibration.

Total flavonoid content (TFC) was quantified using the aluminum chloride colorimetric method [57]. A quercetin stock solution (1000 µg/mL) was prepared in 60% ethanol. For extract preparation, 1000 mg plant tissue was mixed with 50 mL 60% aqueous ethanol, incubated at 30–40 °C for 60 min, filtered, and brought to 100 mL final volume.

For analysis, 2–10 mL of quercetin standards and 0.5 mL sample extract were combined with 2.5 mL 5% sodium nitrite, 2.5 mL 10% AlCl₃, and 20 mL 4% NaOH, then diluted to 50 mL with 60% ethanol. After 15 min, absorbance spectra were recorded using 1.0-cm quartz cuvettes.

### Antioxidant activity assays

DPPH radical scavenging activity was assessed following the method of Brand-Williams et al. [58]. The DPPH reagent (Sigma-Aldrich) was adjusted to an initial absorbance of 0.7–0.8. Reaction mixtures consisted of 50 µL extract, 1.45 mL ethanol, and 0.5 mL DPPH solution. The mixtures were then vortexed and incubated for 15 min. Absorbance was measured at 517 nm. To quantify the scavenging capacity, an ascorbic acid standard curve was generated using serial dilutions from a 2 mM stock solution. The results were expressed as mM ascorbic acid equivalents (mM AsA Eq).

FRAP activity was measured using the method of Benzie and Strain [59]. Leaf tissue (1000 mg) was homogenized in 3 mL methanol, incubated for 1 h, and centrifuged at 13,000 rpm for 10 min. Then, 100 µL supernatant was mixed with 1900 µL FRAP reagent (TPTZ, FeCl₃·6H₂O, acetate buffer; 1:1:10 v/v/v), incubated at 25 °C for 15 min, and read at 593 nm. All measurements were performed in triplicate. For quantification, a standard curve was constructed using serial dilutions of ascorbic acid, and the final FRAP values were expressed as mM ascorbic acid equivalents (mM AsA Eq).

The CUPRAC assay was performed according to Apak et al. [60]. Using the same extract, 100 µL sample was mixed with 1900 µL CUPRAC reagent (CuCl₂·2H₂O, ammonium acetate buffer, and neocuproine; 1:1:1 v/v/v). After 30-min incubation at room temperature, absorbance was recorded at 450 nm. CUPRAC is suitable for evaluating both hydrophilic and lipophilic antioxidants. To quantify the antioxidant capacity, an ascorbic acid standard curve was utilized, and the results were expressed as mM ascorbic acid equivalents (mM AsA Eq).

### Oxidative stress enzyme activities

The activities of APX, SOD, and CAT were determined spectrophotometrically using established protocols.

APX activity was measured at 290 nm based on the oxidation of ascorbate. Leaf tissue (200 mg) was homogenized in 2 mL extraction buffer (0.1 M sodium phosphate, pH 7.0; 0.5 mM ethylenediaminetetraacetic acid [EDTA]; 1 mM ascorbic acid) and centrifuged at 15,000 rpm. The assay mixture contained 50 mM sodium phosphate buffer (pH 7.0), 0.5 mM ascorbic acid, 0.1 mM EDTA, 0.1 mL enzyme extract, and 0.1 mL 0.1% H₂O₂. After 60 min at room temperature, APX activity was calculated using an ascorbic acid standard curve [61].

SOD activity was determined by inhibiting the photochemical reduction of nitroblue tetrazolium (NBT) following Beauchamp and Fridovich [62]. Leaf tissue (200 mg) was homogenized in 0.1 M sodium phosphate buffer (pH 7.0) containing 0.5 mM EDTA and centrifuged at 15,000 rpm. For the assay, 0.1 mL supernatant was added to a reaction mixture containing methionine, NBT, EDTA, phosphate buffer, sodium carbonate, and riboflavin. The mixture was illuminated (75 μmol m⁻^2^ s⁻^1^, 40 W lamp) for 15 min, and absorbance was read at 560 nm. Activity was expressed as % inhibition of NBT reduction.

CAT activity was measured according to Beers and Sizer [63], based on H₂O₂ decomposition. The assay included 0.036% H₂O₂ in 50 mM sodium phosphate buffer (pH 7.0). Three milliliters of this mixture and 100 µL enzyme extract were placed in a quartz cuvette, and absorbance at 240 nm was recorded at 0 and 60 s to calculate CAT activity.

### Oxidative stress indicators

Lipid peroxidation was assessed by measuring MDA. Leaf tissue (500 mg) was homogenized in 10 mL 0.1% trichloroacetic acid (TCA) and centrifuged at 15,000 rpm. One milliliter of supernatant was mixed with 4 mL solution containing 20% TCA and 0.5% thiobarbituric acid (TBA), incubated at 95 °C for 30 min, cooled on ice, and read at 532 and 600 nm. MDA was calculated by subtracting nonspecific absorbance at 600 nm [64].

H₂O₂ content was determined per Alexieva et al. [65]. Leaf tissue (500 mg) was homogenized in 2.5 mL 0.1% TCA and centrifuged at 12,000 rpm. The reaction mixture contained 0.5 mL supernatant, 0.5 mL 100 mM potassium phosphate buffer (pH 7.0), and 2.0 mL 1 M KI. Samples incubated in the dark for 1 h were read at 390 nm, and H₂O₂ concentration was calculated using a standard curve.

### Chlorophyll and carotenoid contents

Chlorophyll a, chlorophyll b, total chlorophyll, and carotenoid contents were quantified according to Arnon [66], while carotenoid content was determined using the equations adapted for 80% acetone by Lichtenthaler [67]. Leaf material (0.1 g) was homogenized in 80% acetone, and absorbance was measured at 663, 645, and 470 nm. For carotenoids, 100 mg tissue was extracted similarly and filtered before reading at 470 nm.

Concentrations were calculated using the following equations:

$$\begin{aligned}&\mathrm{Carotenoids}\ (\mathrm{mg}/\text{g FW}) \\&=\frac{1000{A}_{470}-1.82\text{Chl a}-85.02\text{Chl b}}{198}\\&\times \frac{V}{1000\times g}\end{aligned}$$

$$\begin{aligned}&\text{Chlorophyll a} (\mathrm{mg}/\text{g FW}) \\&=(12.7{A}_{663}-2.69{A}_{645})\times \frac{V}{1000\times g}\end{aligned}$$

$$\begin{aligned}&\text{Chlorophyll b} (\mathrm{mg}/\text{g FW}) \\&=(22.9{A}_{645}-4.68{A}_{663})\times \frac{V}{1000\times g}\end{aligned}$$

$$\begin{aligned}&\text{Total chlorophyll}\left(\frac{\mathrm{mg}}{\mathrm{g}}\mathrm{FW}\right)\\&=\left(20.2{A}_{645}+8.02{A}_{663}\right)\times \frac{V}{1000\times g}\end{aligned}$$

Here, *V* denotes extract volume (mL), *g* denotes tissue fresh weight, and absorbance values correspond to their respective wavelengths.

### Statistical analyses

The experiment followed a factorial arrangement in a completely randomized design (CRD) with three replicates per treatment. Two-way ANOVA was used to evaluate the effects of irrigation regime and NP treatments, including their interactions. When significant differences were detected, Tukey’s HSD test was applied (*p* < 0.05). Analyses were performed using JMP Pro 17. Pearson’s correlation coefficients were calculated and visualized using the corrplot package in R [68]. PCA was conducted to examine relationships among irrigation regimes, treatments, and measured variables using the ggplot2 package [69].

## Results

### Morphological and physiological parameters

**Fig. 3:**
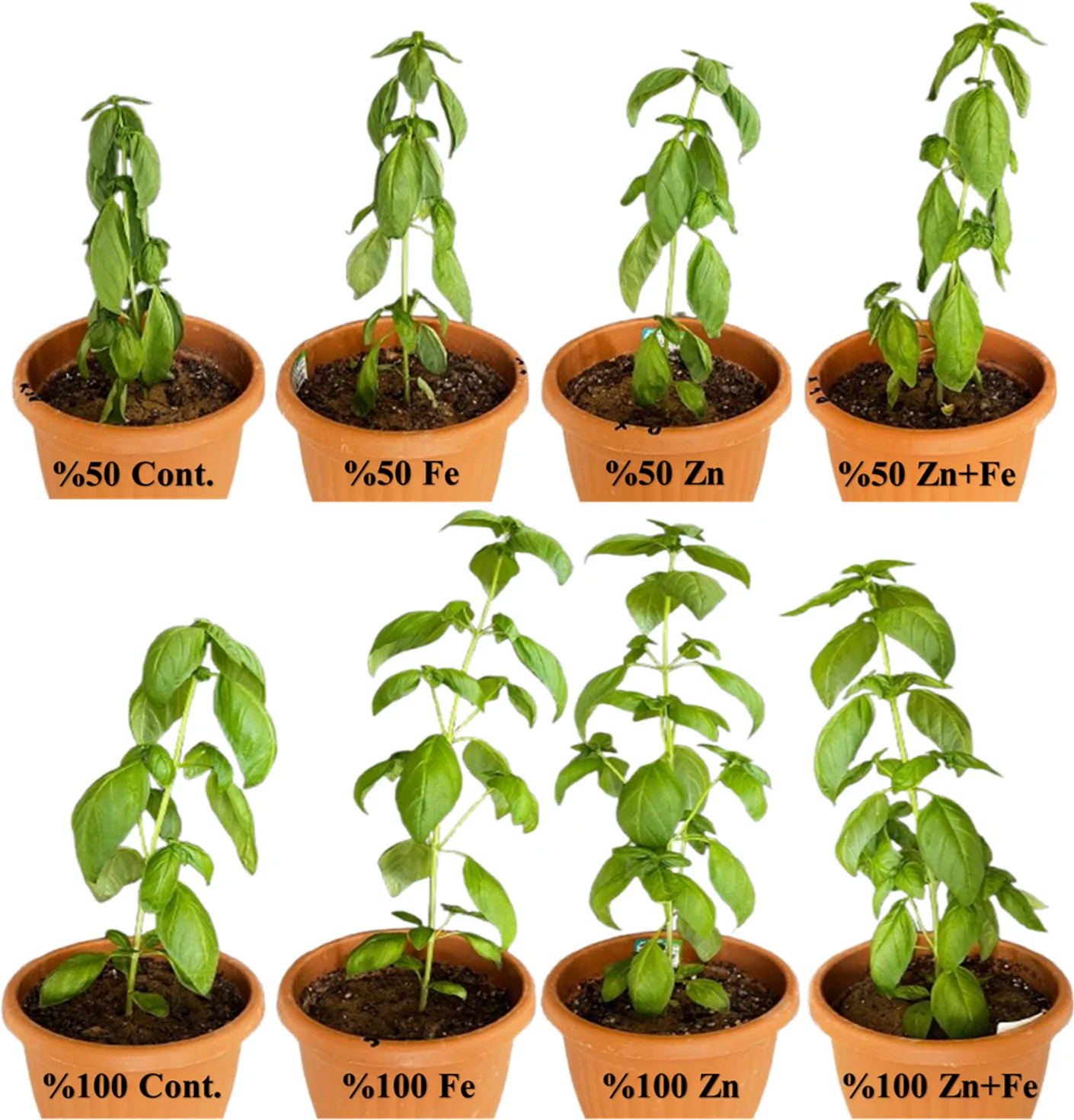
Representative visual appearance of basil plants under different NP treatments and irrigation regimes at the end of the experimental period (Cont.: Control)

**Fig. 4:**
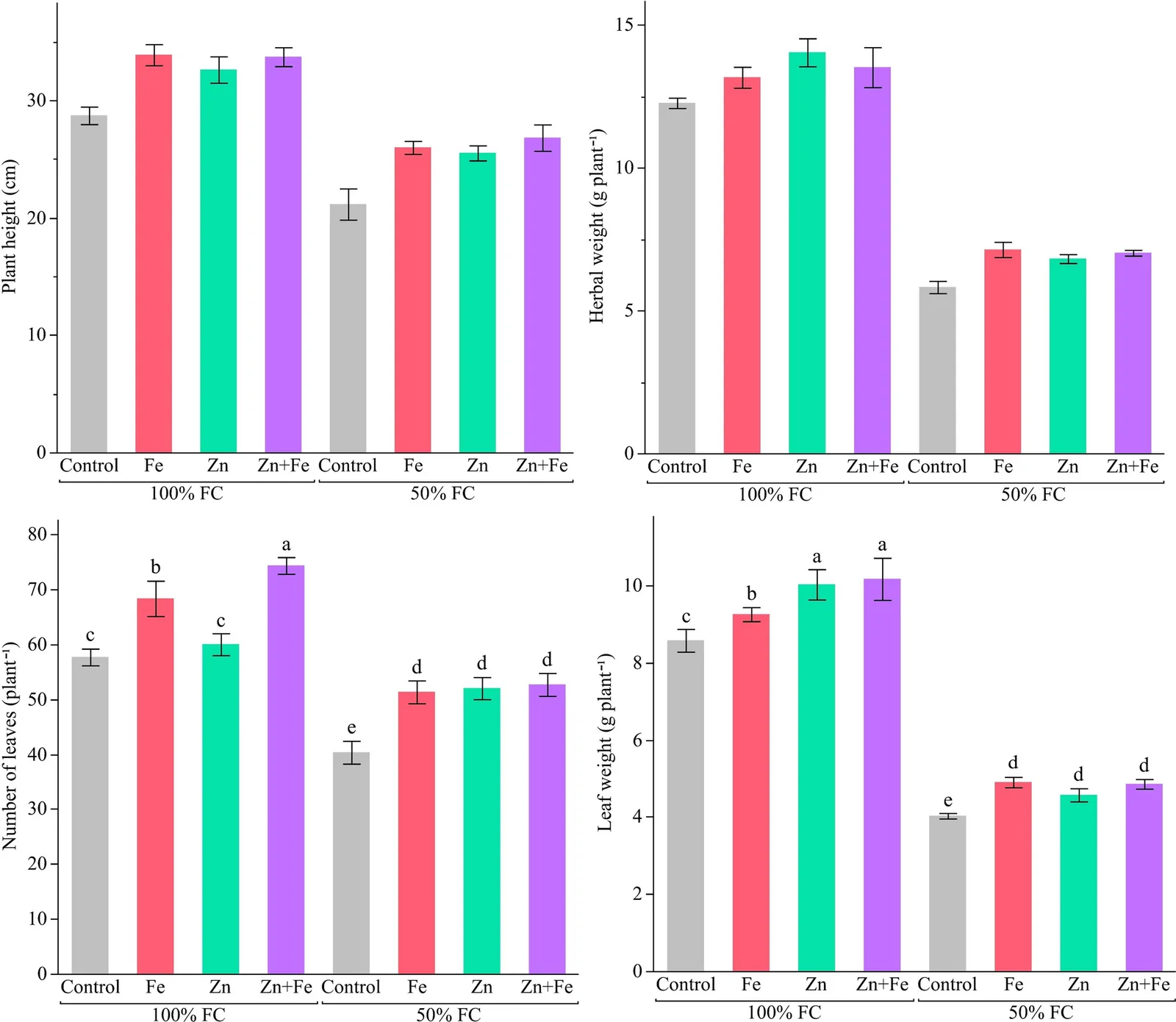
Morphological and physiological responses of basil to green-synthesized Zn, Fe, and Zn + Fe NPs under two irrigation regimes (100% and 50% field capacity), including plant height, number of leaves, fresh herb weight, and fresh leaf weight. Different letters indicate significant differences at *p* < 0.05 (Tukey test)

Nanoparticle treatments and irrigation regimes significantly affected all morphological traits of basil. Across traits, plants at 100% field capacity (FC) performed better than those at 50% FC, confirming the detrimental effect of water limitation (Figs. 3, 4, Table S1). All NP treatments produced taller plants than the control, with Zn + Fe NP giving the highest mean height (30.28 cm), although NP treatments did not differ significantly among themselves (Table S1). The highest leaf number (74.33 leaves plant⁻^1^) was recorded under Zn + Fe NP at 100% FC, significantly exceeding all other combinations. Overall, Zn + Fe NP induced the greatest increase in leaf number, followed by Fe NP and Zn NP, whereas the control had the lowest values. Fresh herb weight was significantly increased by all NPs compared with the control, with Zn NP providing the highest value (14.04 g) under full irrigation. For fresh leaf weight, Zn + Fe NP (10.17 g) gave the highest response, closely followed by Zn NP; both were significantly higher than the control (Table S1, Fig. 4).

### Total phenol and total flavonoid contents

Total phenolic and flavonoid contents responded strongly to both NP treatments and irrigation. The highest TPC (50.08 mg mL⁻^1^ GAE) occurred under Zn + Fe NP at 50% FC, significantly surpassing all other treatments. Under 100% FC, TPC decreased overall; the control (35.18 mg mL⁻^1^ GAE) showed the highest value but did not differ significantly from Zn NP and Fe NP. Across treatment means, Zn NP (38.88 mg mL⁻^1^ GAE) and Zn + Fe NP (38.78 mg mL⁻^1^ GAE) were significantly higher than the control (Table S2).

**Fig. 5:**
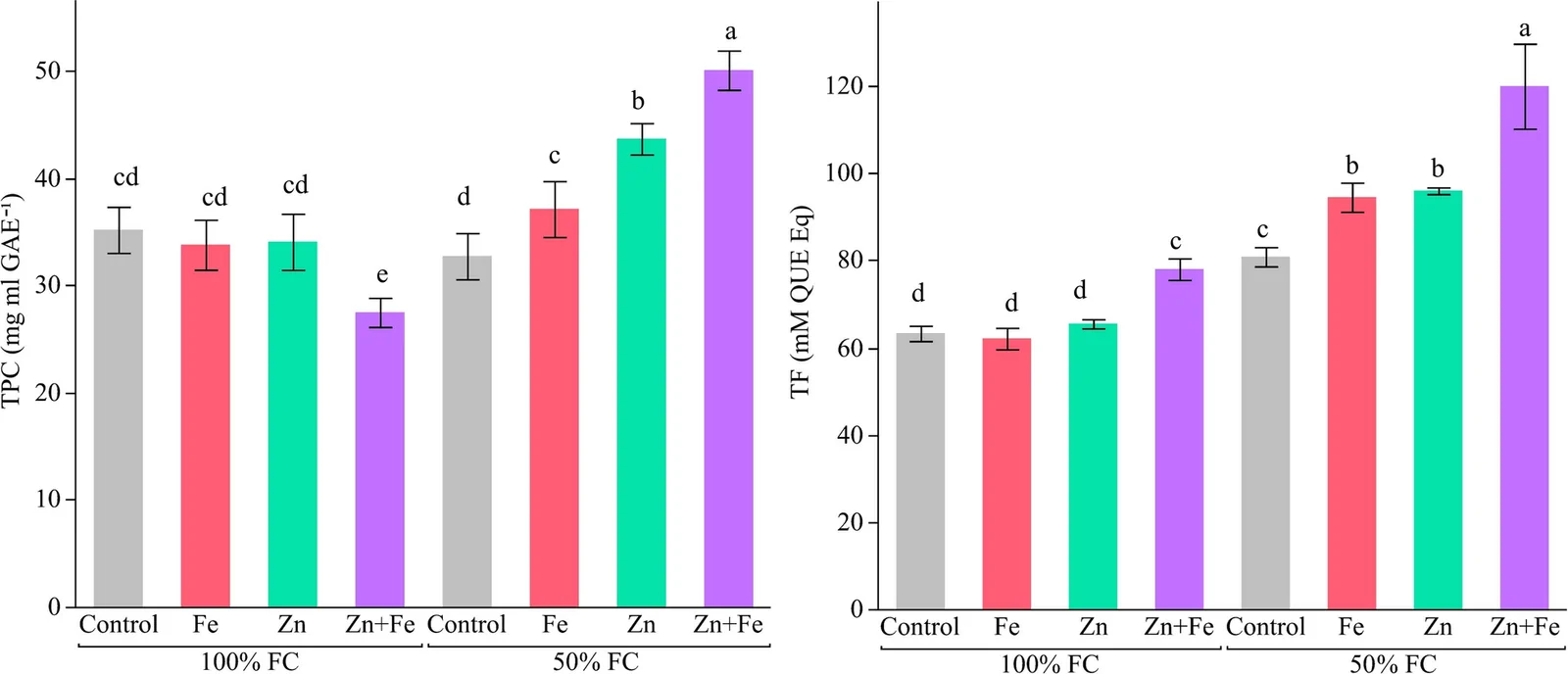
Total phenolic and flavonoid contents of basil as influenced by green-synthesized Zn, Fe, and Zn + Fe NPs under two irrigation regimes (100% and 50% field capacity). Different letters indicate significant differences at *p* < 0.05 (Tukey test)

Similarly, TFC peaked (119.97 mM QUE Eq) under Zn + Fe NP at 50% FC, significantly higher than all other treatment–irrigation combinations. Under full irrigation, flavonoid levels were generally lower, though Zn + Fe NP (78.00 mM) still produced the highest value (Fig. 5). When averaged across irrigation regimes, Zn + Fe NP (98.98 mM) markedly enhanced flavonoids, followed by Zn NP (80.73 mM) and Fe NP (78.29 mM), all exceeding the control (72.06 mM) (Table S2).

### CUPRAC, FRAP, and DPPH activities

**Fig. 6:**
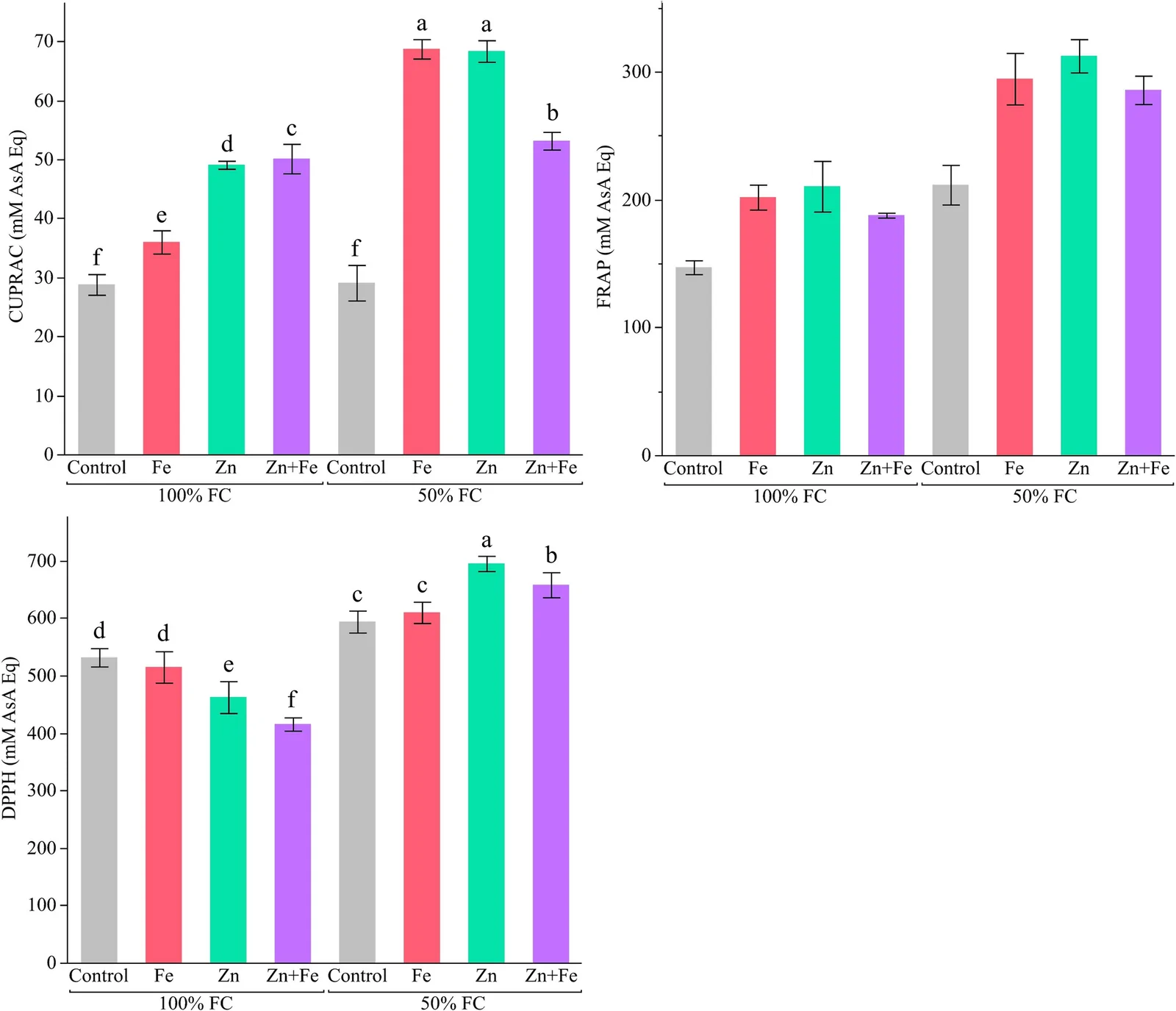
CUPRAC, FRAP, and DPPH antioxidant activities in basil as influenced by green-synthesized Zn, Fe, and Zn + Fe NPs under full (100%) and deficit (50%) irrigation regimes. Different letters indicate statistically significant differences at *p* < 0.05 (Tukey test)

Antioxidant capacities measured by CUPRAC, FRAP, and DPPH showed clear treatment- and irrigation-dependent variation. For CUPRAC, the highest values under 50% FC were recorded with Zn NP (68.34 mM) and Fe NP (68.71 mM), both significantly higher than other treatments (Fig. 6). Under 100% FC, Zn + Fe NP had the greatest CUPRAC value (56.78 mM), followed by Zn NP (49.07 mM) and Fe NP (35.99 mM), whereas the control had the lowest activity (28.79 mM). Averaged across irrigation, Zn NP (58.71 mM) showed the highest CUPRAC capacity, followed by Zn + Fe NP (54.96 mM), Fe NP (52.35 mM), and control (28.93 mM), all significantly different (Table S2).

In the FRAP assay, Zn NP produced the highest mean value (261.45 mM), which was statistically equivalent to Fe NP (248.23 mM). Fe NP also statistically grouped with Zn + Fe NP (236.80 mM), and all nanoparticle applications were significantly above the control (179.32 mM). FRAP activity was also affected by irrigation, with 50% FC (276.10 mM) showing significantly higher values than 100% FC (186.79 mM).

For DPPH radical scavenging, the maximum activity occurred under 50% FC with Zn NP (695.21 mM), significantly higher than all other treatments. Zn + Fe NP also showed high DPPH activity at 50% FC (658.16 mM), whereas the lowest value was obtained under 100% FC with Zn + Fe NP (415.65 mM). Overall, Zn NP (578.89 mM was not significantly different from the control (562.84 mM) and Fe NP (562.48 mM), whereas Zn + Fe NP (536.91 mM) resulted in a significantly lower DPPH mean value.

### CAT, SOD, and APX activities

NP treatments and irrigation regimes markedly influenced antioxidant enzyme activities. For CAT, Zn + Fe NP at 50% FC produced the highest activity (35.67 U mL⁻^1^), while its activity under 100% FC dropped to 3.52 U mL⁻^1^, the lowest among all combinations. When averaged over irrigation regimes, Zn + Fe NP showed the highest CAT activity (19.59 U mL⁻^1^), followed by Zn NP and Fe NP, with the control consistently lowest. Mean CAT activity was significantly higher under 50% FC than 100% FC.

For SOD, Fe NP had the highest mean activity (38.79 U g⁻^1^), statistically grouping with Zn NP (37.42 U g⁻^1^). Zn NP, in turn, shared statistical equivalence with Zn + Fe NP (36.77 U g⁻^1^). Drought conditions increased SOD activity across all treatments, with 50% FC yielding higher means than full irrigation. The control showed the lowest SOD activity in all cases.

**Fig. 7:**
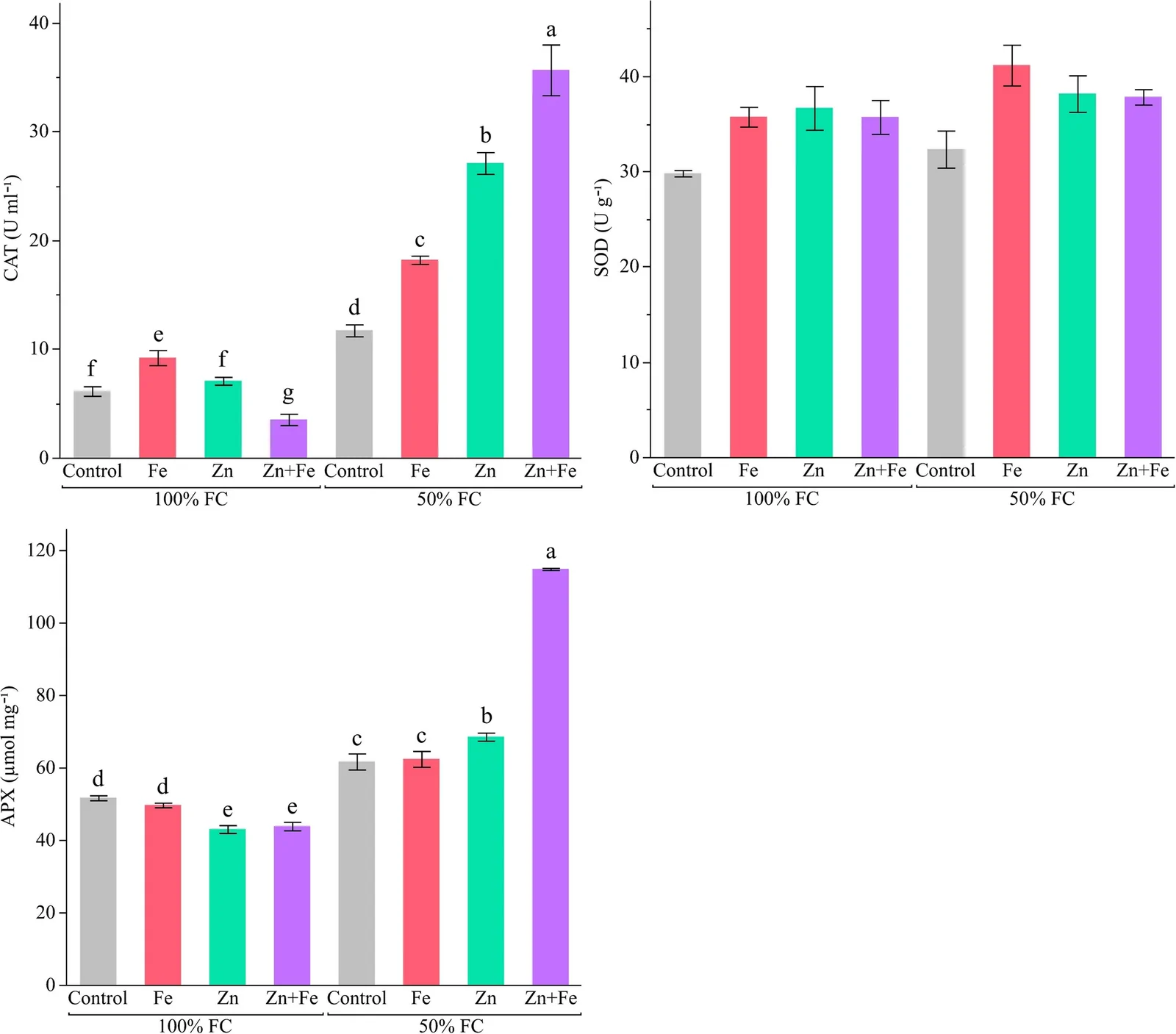
CAT, SOD, and APX enzyme activities in basil in response to green-synthesized Zn, Fe, and Zn + Fe NPs under two irrigation regimes (100% and 50% field capacity). Different letters indicate significant differences at *p* < 0.05 (Tukey test)

APX activity was especially responsive to Zn + Fe NP under drought, reaching 114.92 μmol mg⁻^1^ at 50% FC and clearly surpassing all other treatment–irrigation combinations. In contrast, Zn NP, Fe NP, and the control showed similar APX levels with no significant differences. As with CAT and SOD, APX activity increased to under 50% FC compared with 100% FC. However, Zn + Fe NP at 100% FC resulted in a marked decrease in APX, forming the lowest group within that irrigation regime (Table S3, Fig. 7).

### Oxidative damage indicators

All NP treatments significantly reduced MDA relative to the control, with no significant differences observed among the specific nanoparticle formulations (Fe + Zn, Zn, and Fe, ranging from 7.59 to 8.61 nmol g⁻^1^). The highest MDA accumulation occurred under 50% FC in the control (19.04 nmol g⁻^1^), while the lowest was observed under 100% FC with Fe NP (3.70 nmol g⁻^1^).

**Fig. 8:**
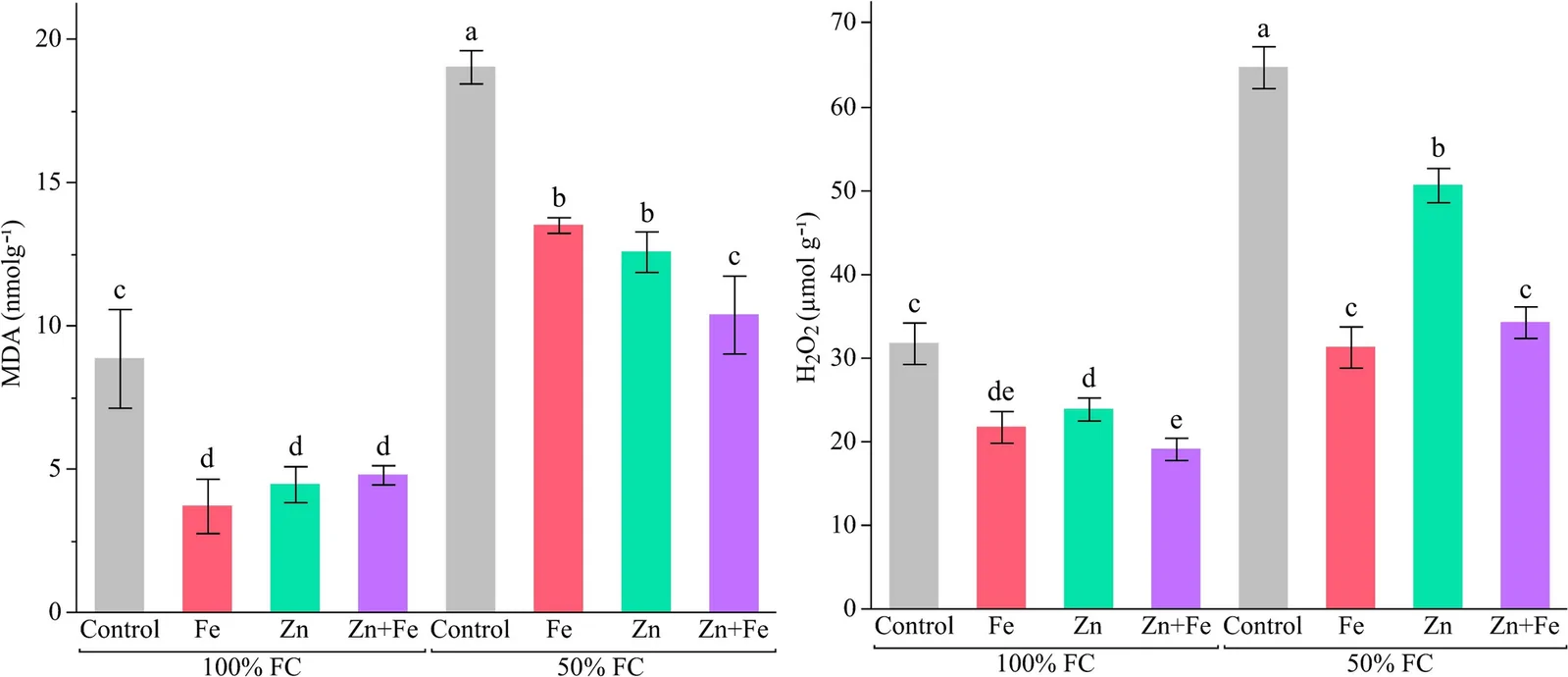
MDA and H₂O₂ contents of basil as affected by green-synthesized Zn, Fe, and Zn + Fe NPs under full (100%) and deficit (50%) irrigation regimes. Different letters denote significant differences at *p* < 0.05 (Tukey test)

H₂O₂ levels also decreased significantly in response to NPs. Under full irrigation, the highest H₂O₂ value among NP treatments (19.05 μmol g⁻^1^) was recorded in Zn + Fe NP, whereas the maximum overall H₂O₂ content occurred in the control at 50% FC (64.66 μmol g⁻^1^). Overall, drought increased both MDA and H₂O₂, but NP applications effectively mitigated this oxidative damage, with Fe NP and Zn + Fe NP demonstrating the most significant and statistically equivalent reductions in mean H₂O₂ levels (Table S3, Fig. 8).

### Chlorophyll and carotenoid contents

**Fig. 9:**
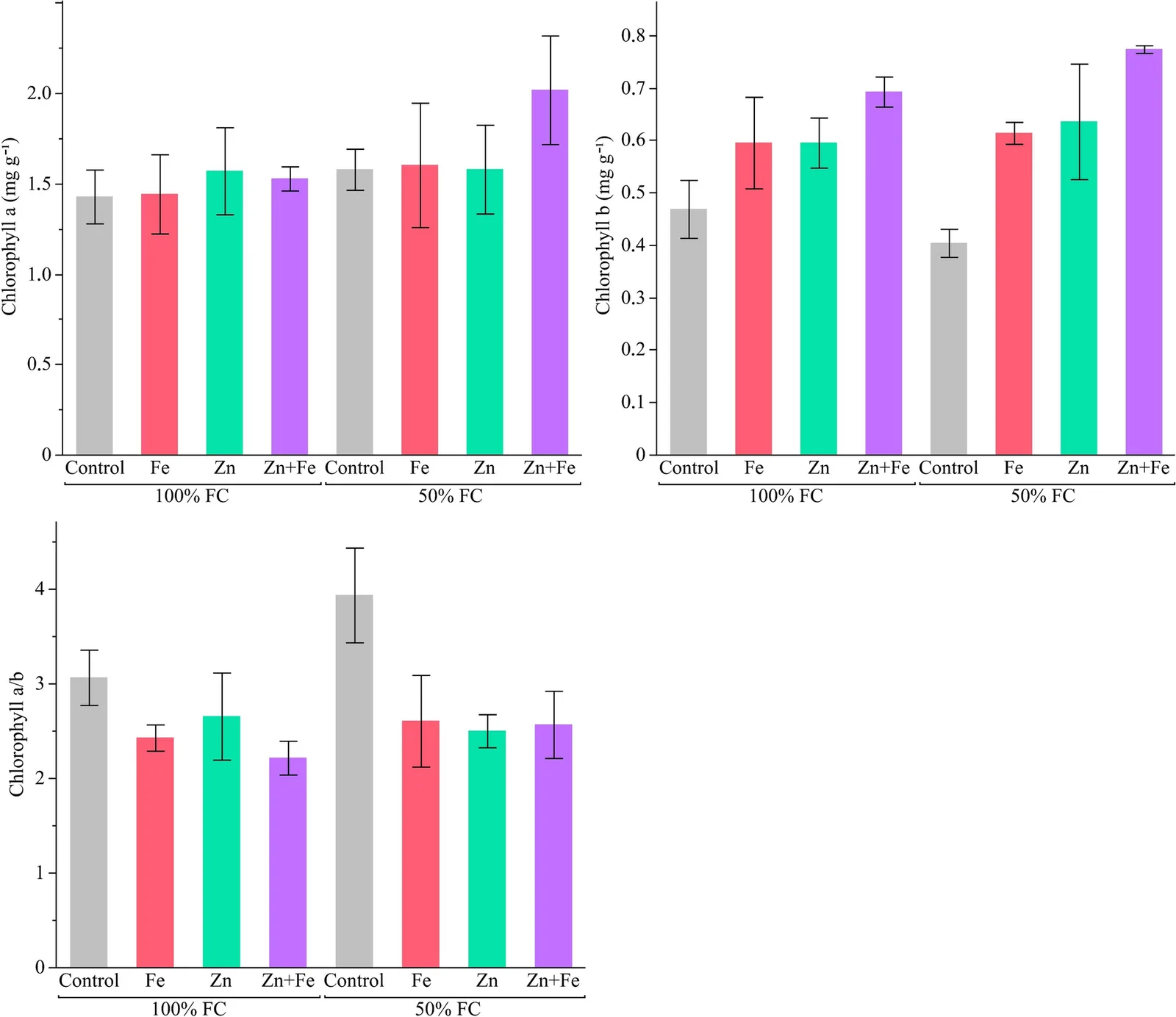
Chlorophyll a, chlorophyll b, total chlorophyll, chlorophyll a/b ratio, and total carotenoid contents of basil as influenced by green-synthesized Zn, Fe, and Zn + Fe NPs under full (100%) and deficit (50%) irrigation regimes

Zn and Fe NP treatments significantly affected chlorophyll and carotenoid contents in basil under drought. All NP applications produced numerically higher chlorophyll a than the control, though differences were not statistically significant. In contrast, chlorophyll b was significantly increased by Zn + Fe NP (0.74 mg g⁻^1^), exceeding the other treatments. The highest chlorophyll a/b ratio (3.49) occurred in the control, suggesting pigment imbalance under stress, whereas NP-treated plants showed more moderate ratios (Table S4, Fig. 9).

**Fig. 10:**
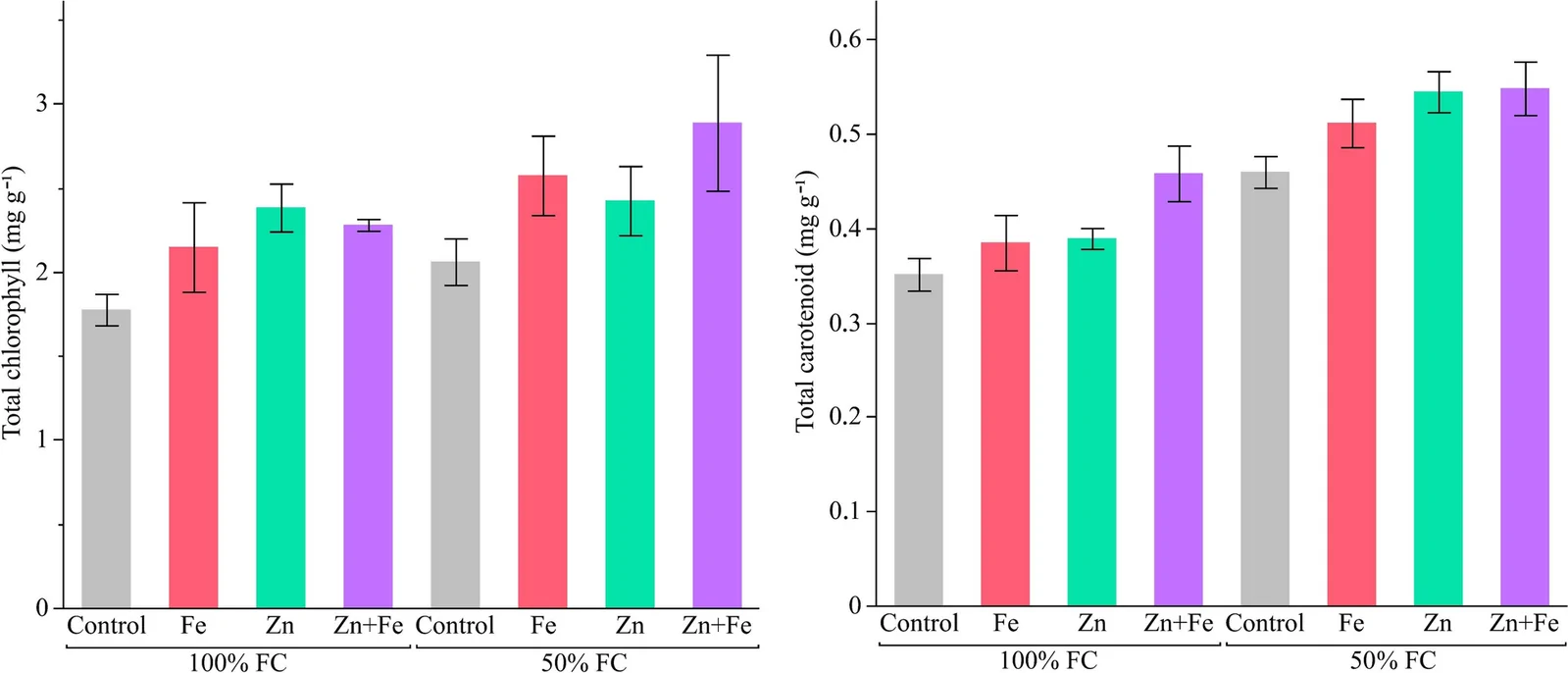
Total chlorophyll and total carotenoid contents of basil as influenced by green-synthesized Zn, Fe, and Zn + Fe NPs under full (100%) and deficit (50%) irrigation regimes

Total chlorophyll was significantly higher in all NP treatments compared with the control, although there were no significant differences among the Zn + Fe, Zn, and Fe NP applications themselves. Total carotenoids were also maximized under Zn + Fe NP (0.50 mg g⁻^1^), which statistically grouped with Zn NP (0.47 mg g⁻^1^). All NP treatments were significantly greater than the control. For all pigment parameters, plants at 50% FC generally showed higher values than those at 100% FC (Table S4, Fig. 10).

### Associations between growth, antioxidant, and pigment-related traits under drought and nanoparticle treatments

**Fig. 11:**
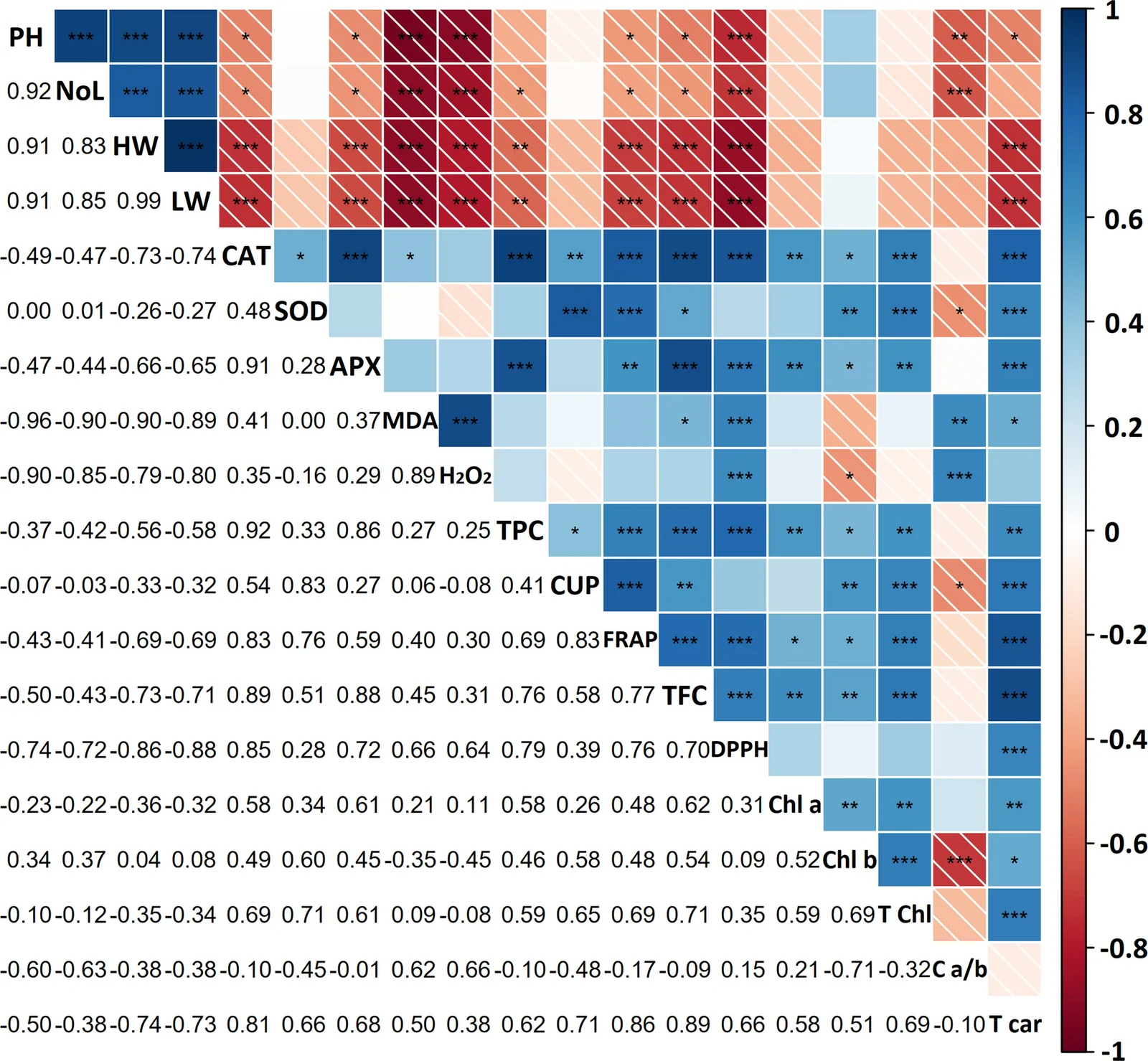
Pearson correlation matrix illustrating relationships among morphological (PH: plant height, NoL: number of leaves, HW: herb weight, LW: leaf weight), antioxidant enzyme (CAT, SOD, APX), oxidative stress (MDA, H₂O₂), phenolic (TPC), flavonoid (TFC), and antioxidant capacity (CUPRAC, FRAP, DPPH) parameters, as well as pigment traits (Chl a: Chlorophyll a, Chl b: Chlorophyll b, C a/b: T Chl: Total Chlorophyll, Chlorophyll a/b, T Car: Total Carotenoid) of basil (*Ocimum basilicum* L.) under two irrigation regimes and nanoparticle treatments. Color intensity represents correlation strength (blue = positive, red = negative). Significance levels: *p* < 0.05 (*), *p* < 0.01 (**), *p* < 0.001 (***)

Correlation analysis revealed clear relationships among morphological, biochemical, and pigment traits in basil under drought and NP treatments (Fig. 11). Strong positive correlations were found among plant height, number of leaves, herb weight, and leaf weight (r = 0.83–0.99, *p* < 0.001), indicating coordinated growth responses to irrigation and NPs. Antioxidant enzymes (CAT, SOD, APX) were positively associated with TPC, TFC, and antioxidant capacities (CUPRAC, FRAP, DPPH), suggesting that enhanced enzymatic activity coincided with greater secondary metabolite accumulation.

Oxidative stress indicators (MDA, H₂O₂) showed strong negative correlations with growth and antioxidant traits (r = − 0.79 to − 0.96, *p* < 0.001), confirming that effective antioxidant activation was linked to reduced lipid peroxidation and accumulation. Pigment variables were positively interrelated; total chlorophyll was closely correlated with carotenoid content, indicating co-regulation of pigment pools under drought and NP treatments.

### Interrelationships among growth, antioxidant activity, and pigment composition of basil under drought and nanoparticle treatments

**Fig. 12:**
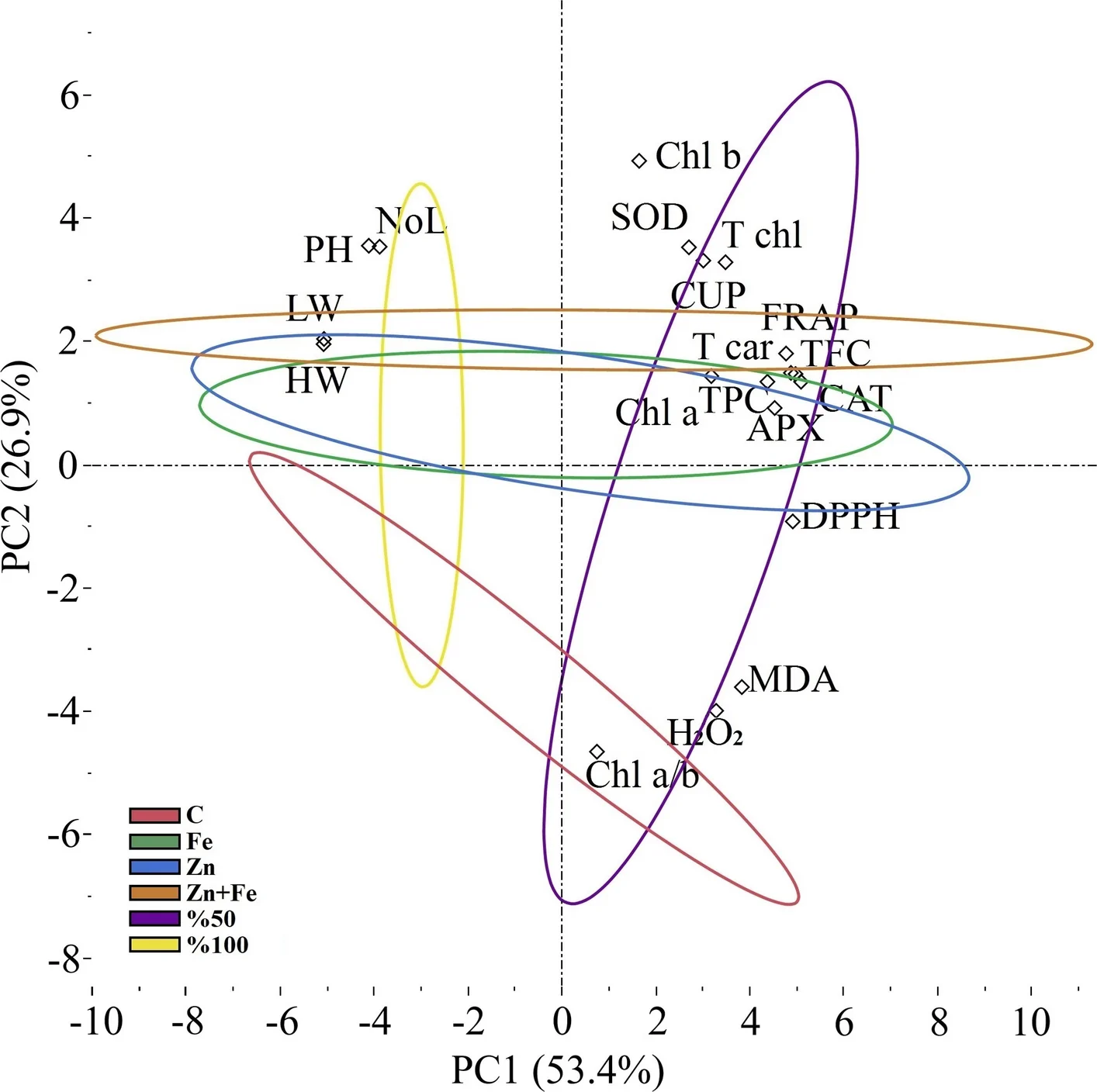
PCA biplot illustrating the distribution of morphological (PH: plant height; HW: herb weight; LW: leaf weight; NoL: number of leaves), antioxidant (CAT, SOD, APX), oxidative stress (MDA, H₂O₂), phenolic (TPC), flavonoid (TFC), antioxidant capacity (CUPRAC, FRAP, DPPH), and pigment parameters (Chl a: Chlorophyll a, Chl b: Chlorophyll b, Chl a/b: T Chl: Total Chlorophyll, Chlorophyll a/b, T Car: Total Carotenoid) of basil (*Ocimum basilicum* L.) subjected to different irrigation regimes and NP applications. Ellipses indicate the 95% confidence regions of each treatment group, corresponding to a significance level of *p* < 0.05

PCA integrated variation in morphological, biochemical, and pigment-related parameters under drought and NP treatments (Fig. 12). PC1 and PC2 accounted for 53.4% and 26.9% of the total variance, respectively, explaining 80.3% of overall variability. The biplot showed clear separation of treatment groups, indicating distinct physiological responses to irrigation regime and NP type.

PC1 represented a gradient from morphological to biochemical traits: plant height, herb weight, leaf weight, and leaf number loaded on the negative side, whereas CAT, APX, TPC, TFC, and antioxidant capacities (CUPRAC, FRAP, DPPH) were located on the positive side. PC2 separated treatments according to oxidative damage and pigment stability. Negative loadings were associated with MDA, H₂O₂, and the chlorophyll a/b ratio, while positive loadings corresponded to SOD, chlorophyll b, CUPRAC, FRAP, and total carotenoids.

Treatment clusters occupied distinct regions of the PCA space. The control group was located in the lower right quadrant near MDA and H₂O₂, whereas Zn + Fe NP clustered within the antioxidant–pigment domain, closely associated with CAT, APX, phenolic and flavonoid contents, and pigment variables. Zn and Fe single NPs appeared in intermediate positions. The 50% FC group was projected toward the biochemical trait region, whereas the 100% FC group clustered with morphological parameters.

## Discussion

The present study demonstrated that both irrigation regimes and nanoparticle applications played a decisive role in modulating basil’s morphological performance. Plants under full irrigation exhibited more vigorous development, likely due to adequate turgor maintenance and sustained cell division [5, 6]. In contrast, drought constrained growth, yet the application of Zn and Fe NPs partly alleviated this limitation. Since statistical analysis indicated no significant differences among the individual nanoparticle treatments for morphological parameters such as plant weight and height, the protective effect appears to stem from a generalized benefit of nano-nutrient supplementation rather than a formulation-specific advantage. This positive effect may be attributed to the fundamental physiological roles of zinc and iron: while Zn regulates auxin metabolism and protein synthesis, Fe is essential for chlorophyll biosynthesis and electron transport during photosynthesis [70, 71]. The nano-scale formulation of these nutrients likely enhanced foliar penetration and translocation within tissues [72, 73], improving bioavailability. While conventional ionic and bulk-metal controls were not included in the present study to isolate the size-effect definitively, based on our morphological evidence (Fig. 1) and current nanotechnology literature, we hypothesize that, the mechanistic distinctiveness of the green-synthesized Zn and Fe nanoparticles used in this study—compared to conventional ionic salts (e.g., ZnSO₄, FeSO₄) or bulk metal oxides—lies in their biogenic nature. Conventional metal salts typically release ions too rapidly in a massive burst, risking localized phytotoxicity and oxidative stress, whereas bulk oxides suffer from poor aqueous solubility and limited stomatal penetration. In contrast, green-synthesized nanoparticles are proposed to be enveloped in a 'phyto-corona'—a capping layer of bioactive secondary metabolites derived from the *Salvia officinalis* extract used during synthesis. This organic capping is thought to not only stabilize the nanoparticles by preventing rapid agglomeration but also facilitate highly efficient, biocompatible cellular internalization. Once inside the tissue, this biogenic coating may allow for a controlled, sustained release of Zn^2^⁺ and Fe^2^⁺ ions, directly matching the plant's metabolic demand without triggering severe heavy-metal toxicity. Furthermore, the residual phytochemicals on the nanoparticle surface may act synergistically with the basil’s endogenous antioxidant network, providing an initial chemical shield that synthetically manufactured or bulk nanoparticles lack.

Similar growth-stimulatory effects of nano-Zn and nano-Fe were demonstrated in coriander (*Coriandrum sativum* L.) [74] and cumin (*Cuminum cyminum* L.) [75] under water-deficit conditions, where increased plant height, biomass, and root development were reported. The increased number of leaves observed under Zn + Fe NP treatment may be linked to synergistic metabolic stimulation [76], while biomass improvements might result from Zn’s role in enzymatic activation and Fe’s participation in cellular energy production pathways [77, 78]. Taken together, these findings indicate a trait-dependent advantage of nanoparticle supplementation rather than a uniform superiority of a single treatment across all growth variables.

Alongside morphological traits, secondary metabolite accumulation was also modulated by environmental conditions and treatments. Drought stimulated the production of phenolic and flavonoid compounds, commonly associated with enhanced antioxidant capacity under abiotic stress [79–81]. The combined application of Zn and Fe NPs further elevated these compounds, possibly due to their involvement as cofactors in biosynthetic pathways of phenolic derivatives [82–85]. Our findings align with those of Kamyab et al. [86] on thyme and Ostadi et al. [87] on peppermint, where Fe-, Zn-, or Ti-based NPs under drought led to elevated phenolics, flavonoids, and antioxidant activity. The antioxidant-capacity assays also supported a strengthened biochemical defense in nanoparticle-treated plants, although the response was assay-dependent rather than uniformly maximal under the combined treatment. The ability of Zn and Fe to stabilize membranes and facilitate antioxidant enzyme function may have contributed to this effect [88–90]. Similar increases in carotenoids, phenols, and flavonoids under nano-Si and nano-Zn were reported for coriander, supporting our observations. Additionally, improved cellular uptake in nano-form may have amplified their physiological effectiveness [34]. Dowom et al. [91] also emphasized the role of chitosan nanoparticles in enhancing soluble sugars, proline, and secondary metabolites under drought, supporting the protective biochemical response triggered by nanoparticles. The increased antioxidant activity observed under drought further underscores the role of secondary metabolites in stress adaptation [92, 93]. Thus, the present results show that the combined treatment was particularly effective in promoting phenolic and flavonoid accumulation, whereas for several antioxidant-capacity endpoints, single-metal nanoparticle applications were statistically equally effective. This stress-specific efficacy is particularly evident in the DPPH assay. While the combined Zn + Fe NP treatment significantly enhanced DPPH scavenging capacity under drought (50% FC) relative to the drought-control, it produced the lowest activity under full irrigation (100% FC), which consequently lowered its overall treatment mean. This counterintuitive response under well-watered conditions suggests that in the absence of severe oxidative stress, the combined metal application does not trigger—and may even slightly downregulate—basal non-enzymatic scavenging pools. Alternatively, it demonstrates that the synergistic biochemical benefit of the Zn + Fe formulation is strictly stress inducible. Ultimately, the nanoparticles provided the critical functional value exactly when required by the plant—under water-deficit conditions. However, these interpretations must be approached with scientific caution. In vitro antioxidant capacity assays (such as DPPH, FRAP, and CUPRAC) primarily measure general chemical reducing potential and may not perfectly capture the intricate in vivo ROS scavenging dynamics. The observed inconsistencies, particularly the striking suppression of DPPH under full irrigation, could also be partially influenced by the specific reaction kinetics of the assay itself or potential chemical interferences from the accumulated metal ions in the specific extract environment. Therefore, while the overarching trend strongly points toward a stress-inducible biochemical defense, the absolute capacity values reflect a complex interplay between the plant's physiological regulation and assay-specific chemical sensitivities, rather than an absolute biological maximum.

Oxidative stress markers such as MDA and H₂O₂ provided further insight into the stress status of the plants. Elevated levels of these markers under drought suggest increased lipid peroxidation and reactive oxygen species accumulation [94, 95]. However, reductions in both MDA and H₂O₂ were noted with nanoparticle applications, particularly Zn + Fe NP. In practical terms, the nanoparticle treatments did not merely accompany the stress response but clearly reduced oxidative damage relative to drought control. As all nanoparticle formulations reduced MDA levels with equal statistical efficiency, the protection of membrane integrity was a shared benefit. Furthermore, both Fe and Zn + Fe NPs provided the most significant and statistically equivalent reductions in H₂O₂. This outcome may be due to the combined ability of Zn and Fe to protect membrane integrity and maintain redox homeostasis [96, 97]. Their role in stimulating key antioxidant enzymes such as SOD, CAT, and APX likely contributed to more efficient detoxification of ROS [98–101]. Similar reductions in oxidative markers and improved enzymatic antioxidant defense were observed with TiO₂ and Si NPs in cumin and coriander under drought stress [74, 75].

The enzymatic antioxidant defense system responded dynamically to both water stress and nanoparticle inputs. CAT and APX activities were particularly sensitive to irrigation regime and treatments. In contrast, SOD activity was enhanced by nanoparticle applications as a main effect, without a significant interaction between irrigation and specific nanoparticle formulations. Zn + Fe NP was especially effective in enhancing CAT and APX, likely due to Zn’s structural stabilization of enzymes and Fe’s catalytic function in redox reactions [102–106]. This pattern points to complementarity among nanoparticle treatments rather than a uniform co-enhancement of all antioxidant enzymes by the combined formulation. Enhanced APX activity, particularly under Zn + Fe NP, reinforces the role of nanoparticle formulations in strengthening enzymatic ROS scavenging mechanisms [107, 108]. This trend parallels findings in *Salvia abrotanoides* by Dowom et al. [91], where chitosan NPs significantly enhanced SOD and peroxidase activity under water deficit. Moreover, the weaker stimulation of some enzyme activities under full irrigation likely reflects lower oxidative demand in well-watered plants, where strong inducible antioxidant activation becomes less necessary. As visually supported by the restricted growth and reduced fresh weight of the 50% FC plants (Fig. 3), relative water content naturally decreases under drought stress. Nevertheless, the substantial rise in specific enzymatic activities (e.g., > 100% increases) far exceeds this proportional loss of tissue water. This mathematical discrepancy clearly points to an active physiological defense mechanism, rather than a simple concentration effect. Furthermore, we hypothesize that the substantial differential in CAT and APX activities between 50% FC and 100% FC (e.g., the transition from 35.67 ± 1.35 to 3.52 ± 0.30 U mL⁻^1^ in Zn + Fe NP treated plants) may reflect a rapid downregulation of these high-cost metabolic pathways in the absence of an oxidative trigger, allowing the plant to reallocate resources toward growth rather than defense. Under water-deficit, the combined Zn + Fe formulation triggered a robust, proactive enzymatic defense response. Conversely, under full irrigation (100% FC), the absence of an oxidative trigger leads to a rapid downregulation of these high-cost metabolic pathways, allowing the plant to reallocate resources toward growth rather than defense.

Photosynthetic pigments were also influenced by water availability and nanoparticle supplementation. In the present experiment, chlorophyll a, total chlorophyll, and carotenoid contents were higher under restricted irrigation than under full irrigation, indicating that pigment responses were not simply suppressed by drought. This pattern may reflect acclimatory adjustment and/or concentration effects associated with reduced tissue water content; therefore, higher pigment concentrations should not be interpreted automatically as evidence of a higher photosynthetic rate [109–111]. Chlorophyll b responded more strongly to nanoparticle treatment, with Zn + Fe NP enhancing its accumulation, likely due to the coordinated roles of Zn in protein folding and Fe in chlorophyll biosynthesis [112, 113]. The chlorophyll a/b ratio also shifted across treatments, but this finding is more cautiously interpreted as a change in pigment balance than as direct evidence of improved light-harvesting efficiency. Total chlorophyll and carotenoid contents were also elevated under drought and further enhanced by nanoparticles, indicating protective roles in photosynthesis and oxidative defense [114–116]. While the combined treatment significantly enhanced chlorophyll b, parameters like total chlorophyll were broadly improved by all nanoparticle treatments without statistically significant superiority of a single formulation. Comparable enhancements in pigment stability under nano-Zn, nano-Fe, and chitosan treatments were also reported in coriander [74] and sage [91], reinforcing the consistency of our findings.

Correlation and multivariate analyses demonstrated coherent modulation of growth, antioxidant defense, and pigment stability in basil under drought and nanoparticle treatments. The strong positive associations among morphological traits indicate coordinated growth responses to treatments improving water-use efficiency and nutrient uptake [117–122]. Likewise, strong positive correlations between antioxidant enzymes (CAT, APX, SOD) and non-enzymatic antioxidants (phenolics, flavonoids, CUPRAC, FRAP, DPPH) show that secondary metabolism and enzymatic defenses operate jointly, consistent with observations from other drought–nanoparticle studies [123]. Conversely, oxidative stress indicators were strongly negatively correlated with both growth and antioxidant capacities, underscoring the role of NP treatments in alleviating drought-induced lipid peroxidation and H₂O₂ accumulation [124, 125]. Pigment traits also correlated positively with antioxidant and growth parameters, reflecting the link between pigment integrity, redox homeostasis, and photosynthetic performance [122]. PCA further highlighted distinct treatment clusters: Zn + Fe NP aligned with antioxidant/pigment domains, single-NP treatments occupied intermediate positions, and control plants grouped with oxidative stress variables. Taking together, these multivariate patterns support the view that nanoparticle treatments improved drought tolerance through coordinated shifts in growth, pigment stability, and redox metabolism rather than through isolated changes in individual traits. These findings agree with broader literature showing the superior performance of multi-micronutrient nanoparticles in promoting stress tolerance [125].

Overall, the correlation and PCA outcomes provide mechanistic support for the interpretation that green-synthesized Zn and Fe NPs enhance drought resilience not by targeting isolated parameters but by reorganizing physiological networks of growth, antioxidant metabolism, and pigment stability. Rather than fully eliminating the usual growth–defense constraint under water deficit, the present data suggest that nanoparticle supplementation helped moderate this trade-off, allowing simultaneous improvement in biomass-related and biochemical defense traits. This coordinated response therefore supports improved biomass retention and quality-related biochemical attributes in medicinal and aromatic plants. Future studies should further examine the molecular signaling, tissue-level nanoparticle fate, direct photosynthetic performance, and long-term ecological implications of such nano-enabled strategies.

## Conclusion

This study established that green-synthesized Zn and Fe NPs serve as potent biostimulants that reorganize basil's physiological network under water-deficit stress. The combined application of Zn + Fe NPs helped moderate the growth-defense trade-off by promoting simultaneous improvements in biomass retention and biochemical defense. Our results indicate that this nano-enabled strategy optimizes the plant's redox homeostasis, evidenced by enhancements of 204% for CAT and 86% for APX activities, alongside a significant reduction in oxidative cellular damage, which collectively ensure superior resilience.

Beyond these findings, the study highlights a practical pathway for field-scale agronomic intervention; the ability of these NPs to stabilize pigment pools and secondary metabolites suggests a realistic potential for maintaining yield and quality in water-limited production systems. However, we explicitly acknowledge that our research serves as a foundational assessment. To transition these findings toward commercial application, future priorities must address current methodological gaps: specifically, the necessity of rigorous material characterization (XRD, FTIR) and the inclusion of ionic and bulk-metal controls. Prioritizing these parameters in follow-up research will be essential to isolate the nano-specific advantages definitively and to establish scalable, dosage-optimized protocols for sustainable crop management.

## Supplementary Information

Supplementary Material 1.

## Data Availability

Data will be made available on request.
