# Supplementary material for: Green-synthesized zinc and iron nanoparticles enhance basil drought tolerance via antioxidant regulation
Source: BMC Plant Biol. 2026 Sep 12;26:1945. doi: 10.1186/s12870-026-09935-3 (PMC13757219; doi:10.1186/s12870-026-09935-3)
Supplement: Supplementary file 1 [file 12870_2026_9935_MOESM1_ESM.docx]

**Green-synthesized zinc and iron nanoparticles enhance basil drought tolerance via antioxidant regulation**

Ibrahim Selvikaya^1^, Rufayi Karataş^2^, Murat Karakuş^3^, Hilal Yilmaz^4^, Fatih Demirel^5^, Emrah Guler^6^, Yusuf Tutar^7^, Abdurrahim Yilmaz^1^*

^1^ Department of Field Crops, Faculty of Agriculture, Bolu Abant Izzet Baysal University, 14030, Bolu, Türkiye; hiselvikaya26@gmail.com, ayilmaz8833@gmail.com

^2^ Faculty of Agriculture, Department of Field Crops, Atatürk University, 25240, Erzurum, Türkiye; rufayik.1@gmail.com

^3^ Department of Plant and Animal Production, Seben	Izzet Baysal Vocational School, Bolu Abant Izzet Baysal University, 14750, Bolu, Türkiye; muratkarakus@ibu.edu.tr

^4^ Plant and Animal Production Program, Izmit Vocational School, Kocaeli University, Kocaeli, 41285, Türkiye; hilal.yilmaz@kocaeli.edu.tr

^5^ Department of Agricultural Biotechnology, Faculty of Agriculture, Igdir University, 76000, Igdir, Türkiye; fatih.demirel@igdir.edu.tr

^6^ Department of Horticulture, Faculty of Agriculture, Bolu Abant Izzet Baysal University, 14030, Bolu, Türkiye; emrahguler@ibu.edu.tr

^7^ Division of Medicinal Biochemistry, Department of Basic Medical Sciences, Faculty of Medicine, Recep Tayyip Erdogan University, 53020, Rize, Türkiye; yusuf.tutar@erdogan.edu.tr

**# Supplementary Material #**

**Table S1.** Comparative Effects of Green-Synthesized Zinc and Iron Nanoparticles under Drought Stress on Morphological and Physiological Traits of Basil

| **Treatments / Parameters** | **Plant height (cm)** | **Herbal weight (g plant^-1^)** | **Leaf weight (g plant^-1^)** | **Number of leaves (plant^-1^)** |
| --- | --- | --- | --- | --- |
| **%50 Control** | 21.17 ± 0.77 | 5.82 ± 0.12 | 4.03 ± 0.04 **e** | 40.33 ± 1.20 **e** |
| **%50 Fe** | 26.00 ± 0.32 | 7.14 ± 0.15 | 4.90 ± 0.08 **d** | 51.33 ± 1.20 **d** |
| **%50 Zn** | 25.53 ± 0.37 | 6.82 ± 0.09 | 4.57 ± 0.10 **d** | 52.00 ± 1.15 **d** |
| **%50 Fe+Zn** | 26.83 ± 0.65 | 7.02 ± 0.06 | 4.85 ± 0.07 **d** | 52.67 ± 1.20 **d** |
| **%100 Control** | 28.73 ± 0.43 | 12.27 ± 0.10 | 8.57 ± 0.17 **c** | 57.67 ± 0.88 **c** |
| **%100 Fe** | 33.90 ± 0.52 | 13.17 ± 0.21 | 10.02 ± 0.11 **a** | 68.33 ± 1.86 **b** |
| **%100 Zn** | 32.63 ± 0.65 | 14.04 ± 0.28 | 9.25 ± 0.23 **b** | 60.00 ± 1.15 **c** |
| **%100 Fe+Zn** | 33.73 ± 0.47 | 13.52 ± 0.41 | 10.17 ± 0.31 **a** | 74.33 ± 0.88 **a** |
| **%50 Mean** | 24.88 ± 0.70 **B** | 6.70 ± 0.16 **B** | 4.59 ± 0.11 **B** | 49.08 ± 1.61 **B** |
| **%100 Mean** | 32.25 ± 0.67 **A** | 13.25 ± 0.23 **A** | 9.50 ± 0.21 **A** | 65.08 ± 2.08 **A** |
| **Control Mean** | 24.95 ± 1.74 **B** | 9.05 ± 1.44 **B** | 6.30 ± 1.02 **C** | 49.00 ± 3.93 **D** |
| **Fe Mean** | 29.95 ± 1.79 **A** | 10.15 ± 1.35 **A** | 7.08 ± 0.94 **B** | 59.83 ± 3.92 **B** |
| **Zn Mean** | 29.08 ± 1.62 **A** | 10.43 ± 1.62 **A** | 7.30 ± 1.23 **AB** | 56.00 ± 1.93 **C** |
| **Fe+Zn Mean** | 30.28 ± 1.58 **A** | 10.27 ± 1.46 **A** | 7.51 ± 1.20 **A** | 63.50 ± 4.89 **A** |

**Fe**: Iron nanoparticle application, **Zn**: Zinc nanoparticle application, **Fe+Zn**: Iron and Zinc mixed nanoparticle application,

**%50**: Half irrigation, **%100**: Full irrigation,

Different letters in the same column indicate significant differences at *p <* 0.05 according to the Tukey test.

**Table S2.** Comparative Effects of Green-Synthesized Zinc and Iron Nanoparticles under Drought Stress on Antioxidant Capacity Analysis of Basil

| **Treatments / Parameters** | **Total phenolic content (mg ml GAE^-1^)** | **Total flavonoid content (mM QUE Eq)** | **CUPRAC (mM QUE Eq)** | **FRAP (mM QUE Eq)** | **DPPH (mM QUE Eq)** |
| --- | --- | --- | --- | --- | --- |
| **%50 Control** | 32.74 ± 1.25 **d** | 80.84 ± 1.28 **c** | 29.07 ± 1.74 **f** | 211.57 ± 8.95 | 594.00 ± 11.03 **c** |
| **%50 Fe** | 37.13 ± 1.51 **c** | 94.48 ± 1.93 **b** | 68.71 ± 0.95 **a** | 294.57 ± 11.66 | 610.00 ± 10.72 **c** |
| **%50 Zn** | 43.68 ± 0.84 **b** | 95.99 ± 0.45 **b** | 68.34 ± 1.06 **a** | 312.47 ± 7.51 | 695.21 ± 7.62 **a** |
| **%50 Fe+Zn** | 50.08 ± 1.06 **a** | 119.97 ± 5.63 **a** | 53.14 ± 0.87 **c** | 285.80 ± 6.42 | 658.16 ± 12.60 **b** |
| **%100 Control** | 35.18 ± 1.24 **cd** | 63.27 ± 1.02 **d** | 28.79 ± 1.01 **f** | 147.07 ± 3.15 | 531.68 ± 9.21 **d** |
| **%100 Fe** | 33.80 ± 1.35 **cd** | 62.09 ± 1.42 **d** | 35.99 ± 1.14 **e** | 201.89 ± 5.65 | 514.95 ± 15.84 **d** |
| **%100 Zn** | 34.07 ± 1.51 **cd** | 65.47 ± 0.61 **d** | 49.07 ± 0.39 **d** | 210.43 ± 11.43 | 462.57 ± 16.05 **e** |
| **%100 Fe+Zn** | 27.49 ± 0.78 **e** | 78.00 ± 1.41 **c** | 56.78 ± 1.45 **b** | 187.79 ± 1.13 | 415.65 ± 6.66 **f** |
| **%50 Mean** | 40.91 ± 2.05 **A** | 97.82 ± 4.44 **A** | 54.82 ± 4.89 **A** | 276.10 ± 12.20 **A** | 639.34 ± 12.88 **A** |
| **%100 Mean** | 32.64 ± 1.05 **B** | 67.21 ± 1.98 **B** | 42.66 ± 2.75 **B** | 186.79 ± 7.85 **B** | 481.21 ± 14.78 **B** |
| **Control Mean** | 33.96 ± 0.96 **B** | 72.06 ± 4.00 **C** | 28.93 ± 0.90 **D** | 179.32 ± 15.03 **C** | 562.84 ± 15.35 **A** |
| **Fe Mean** | 35.47 ± 1.17 **B** | 78.29 ± 7.32 **B** | 52.35 ± 7.35 **C** | 248.23 ± 21.52 **AB** | 562.48 ± 22.91 **A** |
| **Zn Mean** | 38.88 ± 2.28 **A** | 80.73 ± 6.83 **B** | 58.71 ± 4.34 **A** | 261.45 ± 23.63 **A** | 578.89 ± 52.62 **A** |
| **Fe+Zn Mean** | 38.78 ± 5.09 **A** | 98.98 ± 9.74 **A** | 54.96 ± 1.01 **B** | 236.80 ± 22.11 **B** | 536.91 ± 54.60 **B** |

**Fe**: Iron nanoparticle application, **Zn**: Zinc nanoparticle application, **Fe+Zn**: Iron and Zinc mixed nanoparticle application,

**%50**: Half irrigation, **%100**: Full irrigation,

Different letters in the same column indicate significant differences at *p <* 0.05 according to the Tukey test.

**Table S3.** Comparative Effects of Green-Synthesized Zinc and Iron Nanoparticles under Drought Stress on Stress Indicators of Basil

| **Treatments / Parameters** | **CAT (U ml-¹)** | **SOD (U g-¹)** | **APX (µmol mg-¹)** | **MDA (nmolg-¹)** | **H_2_O_2_ (µmol g-¹)** |
| --- | --- | --- | --- | --- | --- |
| **%50 Control** | 11.72 ± 0.32 **d** | 32.35 ± 1.13 | 61.71 ± 1.28 **c** | 19.04 ± 0.33 **a** | 64.66 ± 1.44 **a** |
| **%50 Fe** | 18.20 ± 0.22 **c** | 41.17 ± 1.24 | 62.44 ± 1.26 **c** | 13.51 ± 0.16 **b** | 31.22 ± 1.42 **c** |
| **%50 Zn** | 27.10 ± 0.58 **b** | 38.17 ± 1.11 | 68.56 ± 0.64 **b** | 12.58 ± 0.41 **b** | 50.58 ± 1.18 **b** |
| **%50 Fe+Zn** | 35.67 ± 1.35 **a** | 37.83 ± 0.47 | 114.92 ± 0.17 **a** | 10.38 ± 0.79 **c** | 34.19 ± 1.09 **c** |
| **%100 Control** | 6.13 ± 0.26 **f** | 29.80 ± 0.19 | 51.70 ± 0.39 **d** | 8.85 ± 1.00 **c** | 31.68 ± 1.43 **c** |
| **%100 Fe** | 9.21 ± 0.40 **e** | 36.67 ± 0.59 | 49.70 ± 0.37 **d** | 3.70 ± 0.55 **d** | 21.68 ± 1.10**de** |
| **%100 Zn** | 7.08 ± 0.21 **f** | 35.74 ± 1.33 | 43.05 ± 0.62 **e** | 4.46 ± 0.36 **d** | 23.81 ± 0.79 **d** |
| **%100 Fe+Zn** | 3.52 ± 0.30 **g** | 35.72 ± 1.02 | 43.87 ± 0.66 **e** | 4.79 ± 0.19 **d** | 19.05 ± 0.77 **e** |
| **%50 Mean** | 23.17 ± 2.75 **A** | 37.38 ± 1.05 **A** | 76.91 ± 6.68 **A** | 13.88 ± 0.98 **A** | 45.16 ± 4.09**A** |
| **%100 Mean** | 6.48 ± 0.63 **B** | 34.65 ± 0.91 **B** | 47.08 ± 1.14 **B** | 5.45 ± 0.65 **B** | 24.06 ±1.49 **B** |
| **Control Mean** | 8.93 ± 1.26 **D** | 31.07 ± 0.75 **C** | 56.70 ± 2.32 **B** | 13.95 ± 2.33 **A** | 48.17 ± 7.43 **A** |
| **Fe Mean** | 13.71 ± 2.02 **C** | 38.79 ± 1.36 **A** | 56.07 ± 2.91 **B** | 8.61 ± 2.21 **B** | 26.45 ± 2.28 **C** |
| **Zn Mean** | 17.09 ± 4.49 **B** | 37.42 ± 0.84 **AB** | 55.80 ± 5.71 **B** | 8.52 ± 1.83 **B** | 37.19 ± 6.02 **B** |
| **Fe+Zn Mean** | 19.59 ± 7.21 **A** | 36.77 ± 0.69 **B** | 79.40 ± 15.89 **A** | 7.59 ± 1.30 **B** | 26.62 ± 3.44 **C** |

**Fe**: Iron nanoparticle application, **Zn**: Zinc nanoparticle application, **Fe+Zn**: Iron and Zinc mixed nanoparticle application,

**%50**: Half irrigation, **%100**: Full irrigation,

Different letters in the same column indicate significant differences at *p <* 0.05 according to the Tukey test.

**Table S4.** Comparative Effects of Green-Synthesized Zinc and Iron Nanoparticles under Drought Stress on Photosynthetic Pigment Analysis of Basil

| **Treatments / Parameters** | **Chlorophyll a (mg g^-^¹)** | **Chlorophyll b (mg g^-^¹)** | **Chlorophyll a/b** | **Total chlorophyll (mg g^-^¹)** | **Total carotenoid (mg g^-^¹)** |
| --- | --- | --- | --- | --- | --- |
| **%50 Control** | 1.58 ± 0.07 | 0.40 ± 0.02 | 3.93 ± 0.29 | 2.06 ± 0.08 | 0.46 ± 0.01 |
| **%50 Fe** | 1.60 ± 0.20 | 0.61 ± 0.01 | 2.60 ± 0.28 | 2.57 ± 0.14 | 0.51 ± 0.02 |
| **%50 Zn** | 1.58 ± 0.14 | 0.64 ± 0.06 | 2.50 ± 0.10 | 2.42 ± 0.12 | 0.55 ± 0.01 |
| **%50 Fe+Zn** | 2.02 ± 0.17 | 0.77 ± 0.01 | 2.56 ± 0.20 | 2.89 ± 0.23 | 0.55 ± 0.02 |
| **%100 Control** | 1.43 ± 0.09 | 0.47 ± 0.03 | 3.06 ± 0.17 | 1.78 ± 0.05 | 0.35 ± 0.01 |
| **%100 Fe** | 1.44 ± 0.13 | 0.60 ± 0.05 | 2.42 ± 0.08 | 2.15 ± 0.15 | 0.39 ± 0.02 |
| **%100 Zn** | 1.57 ± 0.14 | 0.60 ± 0.03 | 2.65 ± 0.27 | 2.38 ± 0.08 | 0.39 ± 0.01 |
| **%100 Fe+Zn** | 1.53 ± 0.04 | 0.69 ± 0.02 | 2.21 ± 0.10 | 2.28 ± 0.02 | 0.46 ± 0.02 |
| **%50 Mean** | 1.69 ± 0.09 **A** | 0.61 ± 0.04 | 2.90 ± 0.21 **A** | 2.49 ± 0.11 **A** | 0.52 ± 0.01 **A** |
| **%100 Mean** | 1.49 ± 0.05 **B** | 0.59 ± 0.03 | 2.59 ± 0.12 **B** | 2.15 ± 0.08 **B** | 0.40 ± 0.01 **B** |
| **Control Mean** | 1.50 ± 0.06 | 0.44 ± 0.02 **C** | 3.49 ± 0.25 **A** | 1.92 ± 0.08 **B** | 0.41 ± 0.03 **C** |
| **Fe Mean** | 1.52 ± 0.11 | 0.60 ± 0.02 **B** | 2.51 ± 0.14 **B** | 2.36 ± 0.13 **A** | 0.45 ± 0.03 **B** |
| **Zn Mean** | 1.58 ± 0.09 | 0.62 ± 0.03 **B** | 2.57 ± 0.13 **B** | 2.40 ± 0.07 **A** | 0.47 ± 0.04 **AB** |
| **Fe+Zn Mean** | 1.77 ± 0.14 | 0.74 ± 0.02 **A** | 2.39 ± 0.13 **B** | 2.58 ± 0.17 **A** | 0.50 ± 0.02 **A** |

**Fe**: Iron nanoparticle application, **Zn**: Zinc nanoparticle application, **Fe+Zn**: Iron and Zinc mixed nanoparticle application,

**%50**: Half irrigation, **%100**: Full irrigation,

Different letters in the same column indicate significant differences at *p <* 0.05 according to the Tukey test.
